# Expression of Proton-Sensitive GPR31, GPR151, TASK1 and TASK3 in Common Skin Tumors

**DOI:** 10.3390/cells11010027

**Published:** 2021-12-23

**Authors:** Antonia Förch, Susanne Wallner, Florian Zeman, Tobias Ettl, Christoph Brochhausen, Stephan Schreml

**Affiliations:** 1 Department of Dermatology, University Medical Center Regensburg, Franz-Josef-Strauß-Allee 11, 93053 Regensburg, Germany; antonia.foerch@stud.uni-regensburg.de (A.F.); susanne.wallner@ukr.de (S.W.); 2 Center for Clinical Studies, University Medical Center Regensburg, Franz-Josef-Strauß-Allee 11, 93053 Regensburg, Germany; florian.zeman@ukr.de; 3 Department of Maxillofacial Surgery, University Medical Center Regensburg, Franz-Josef-Strauß-Allee 11, 93053 Regensburg, Germany; tobias.ettl@ukr.de; 4 Institute of Pathology, University of Regensburg, Franz-Josef-Strauß-Allee 11, 93053 Regensburg, Germany; christoph.brochhausen@ukr.de

**Keywords:** GPCR31/151, TASK1/3, Task1/3, skin tumors

## Abstract

TWIK-related acid-sensitive potassium channels TASK1 and TASK3, as well as the G-protein-coupled receptors GPR31 and GPR151, are proton-sensitive membrane proteins. They can be activated or inhibited by low extracellular pH (pHe), which is a hallmark of the tumor microenvironment in solid tumors. However, the role of these channels in the development of skin tumors is still unclear. In this study, we investigated the expression profiles of TASK1, TASK3, GPR31 and GPR151 in squamous cell carcinomas (SCCs), basal cell carcinomas (BCCs), nevus cell nevi (NCN), and malignant melanomas (MMs). We performed immunohistochemistry using paraffin-embedded tissue samples from patients and found that most skin tumors express TASK1/3 and GPR31/151. The results show that BCCs are often negative for GPR31/151 as well as for TASK1/3, while nearly all SCCs express these markers. MMs and NCN show similar expression patterns. However, some tumors show a decreasing TASK1/3 expression in deeper dermal tumor tissue, while GPCRs were expressed more evenly. The lower frequency of GPR31/151 and TSAK1/3 expression in BCCs when compared to SCCs is a novel histological feature distinguishing these two entities. Moreover, BCCs also show lower expression of GPR31/151 and TASK1/3 as compared to NCN and MMs.

## 1. Introduction

The inverse pH gradient (extracellular pHe < intracellular pHi) is a hallmark of solid tumor cells [1]. Acidic metabolic waste products in the tumor microenvironment (TME) result from poor blood perfusion with subsequent hypoxia as well as from inflammation and high metabolic activity [1,2]. H+ ions and lactate accumulate in the TME due to metabolic changes in tumors as well as the altered activity/expression of membrane-bound transporters. The decrease in pHe activates proton-sensitive receptors, such as certain G-protein coupled receptors (GPCRs), transient receptor potential channels (TRPCs), acid-sensing ion channels (ASICs) as well as TWIK-related acid-sensitive potassium channels (TASKs) [3]. Furthermore, tumor cells exhibit complex pH regulation via sodium hydrogen exchanger-1 (NHE1), monocarboxylate transporters (MCT1-4), bicarbonate transporters, vacuolar ATPases (VATPase) and carboanhydrases (CAII, CAIX, CAXII) [1,4,5,6]. As a result, cancer cells exhibit a more alkaline pHi > 7.2 and correspondingly a lower pHe of 6.7–7 compared to normal cells [2]. It is remarkable that tumor cells regulate their intra- to extracellular pH gradient in a very small range, meaning that they not only prevent acidosis-induced apoptosis, but also take advantage by inhibiting the innate immune system [7,8,9], which also enhances tumor growth and metastasis [10].

The heptahelical GPCRs regulate multiple intracellular signaling cascades and therefore are used as a target for more than 50% of the currently registered drugs [11]. Among the ~800 identified GPCRs, only a few are involved in sensing pH changes through the protonation of hydrogen bonds between histidine residues, i.e., GPR4, GPR65, GPR68 and GPR132 [12,13]. This conformational change leads to the activation of the heterodimeric G-protein, which then binds and activates numerous downstream effector proteins. Recently, two more pH-sensitive GPCRs have been reported, GPR31 and GPR151 [14,15,16].

GPR31 has been identified as a target receptor for 12(S)-hydroxyeicosatetraenoic acid (12(S)-HETE) [16], and therefore shares the property of being a lipid receptor with the other proton-sensing GPCRs. GPR31 was identified to mediate KRAS membrane association and is crucial for the proliferation and survival of KRAS-dependent tumors, suggesting that GPR31 may represent a target for anti-RAS therapy [17]. GPR31 has shown the highest protein identity (up to 36%) with hydroxycarbocylic acid receptors (HCAR) 1–3 and becomes activated under acidic conditions. As HCARs play a role in skin cancer and epidermal differentiation, GPR31 could also be involved in skin tumor development.

GPR151 is an orphan GPCR, which means that its endogenous ligand remains unknown. This receptor plays a role in the central nervous system of vertebrates [18]. In contrast to other proton-sensing GPCRs, such as GPR4, GPR65 and GPR68, GPR151 is coupled with Gi, meaning it is a blocking adenylate cyclase [14,19,20]. Its structure contains at least three residues, which become protonated under acidic conditions. As a result, the activated GTPyS can drive changes in gene expression, and also by coordinating phosphorylation and sensitization of TRP channels [14].

Two-pore potassium channels (K2P) consist of four transmembrane domains (TM1-4), two pore-forming domains (P1-2) and an extracellular cap between TM1 + P1, assembling as either homo- or heterodimers [21,22,23]. Its main function is to set the resting membrane potential and therefore the regulation of cellular excitability. There are multiple triggering factors, e.g., light, temperature, decreased pH or membrane stretching [24,25]. To date, there are 15 different subtypes, grouped into six subclasses (TWIK, TREK, TRASK, TALK, THIK, TRESK), in the K2P family [3,25].

TWIK-related acid-sensitive potassium channel TASK1 (encoded by potassium two-pore domain channel subfamily K member KCNK3 gene) as well as TASK3 (encoded by KCNK9) are markedly proton-sensitive and are inhibited as pHe decreases below physiological values. Hence, K^+^ efflux is reduced, which contributes to the depolarization of the cell. Protonation takes place on the H98 residue, but other residues in the M1-P1 loop must exist, as many experiments show [26]. Both channels are expressed in neurons and are responsible for acid-induced nociception, as well as thermosensation. In terms of cancer, TASK1 has been reported to play a role in NSCSL [27]. TASK3 is frequently amplified in various tumors [28], and it seems to foster proliferation and migration [29].

## 2. Materials and Methods

For our experiments, we used paraffin-embedded tissue samples from the dermatopathological routine of the Department of Dermatology, University Medical Center Regensburg. All samples were older than 10 years and therefore free to use under German legislation. An overview of basic patient data (non-identifiable) can be found in Supplementary Table S1.

### 2.1. Immunohistochemistry

Tissue samples (embedded and fixed in paraffin) were cut into 3 µm-thick sections using a microtome and then fixated on slides. Each slide was also stained with hematoxylin and eosin. This and all other subsequent staining steps were performed at room temperature. To minimize differences in staining of the four different tumor subtypes, we conducted each antibody staining step on every tumor entity within two days.

We removed paraffin from the tissue sections by incubating them for 60 min at 72 °C, and then we rehydrated the slides with decreasing alcohol concentrations as follows: 3 × xylol for 10 min, 2 × 100% ethanol for 5 min, 2 × 96% ethanol for 5 min, 2 × 70% ethanol for 5 min. To prevent false-positive results, endogenous peroxidase was blocked with 3% H_2_O_2_ (Fisher Scientific, No. 1404697) for 10 min. Simultaneously, an acidic citrate buffer with pH 6 (Zytomed, Bargteheide, Germany, REF ZUC028) was boiled for 30 min. The slides were washed in distillated water and then boiled for 20 min in the precooked citrate buffer, followed by cooling on ice for 20 min. Subsequently, they were transferred to PBS (Sigma-Aldrich, Darmstadt, Germany, No. D8537) for 10 min. Afterwards, slides were fixed in cover slides and once again washed with PBS. To avoid unspecific antibody binding, proteins were blocked with blocking solution (ZytoChem Plus HRP Kit/Rabbit, Zytomed, Bargteheide, Germany, REF HRP060-Rb) for 10 min. Tissue sections were incubated with the primary rabbit anti-human GPR31 (1:2000 Anti-GPR31 antibody Abcam, Cambridge, UK, ab75579) GPR151 (1:400;Anti-GPCR GPR151 antibody Life Technologies, Waltham, MA, USA Cat.Nr.PA532803), TASK1 (1:666 Anti-TASK1/TASK1 Abcam, Cambridge, UK, Cat.Nr. ab238588;), TASK3 (1:500 Anti-TASK3/Task3 LSBio, Seattle, WA, USA LS_C406505/186347) polyclonal antibodies at 4 °C overnight.

The following day, the slides were washed three times with PBS. The tissue sections were then incubated with the secondary biotinylated antibody for 30 min, they were washed again three times with PBS, then incubated with streptavidin-HRP-conjugate for 20 min and washed 3× with PBS. Positive controls were stained with AEC plus (Dako, Santa Clara, CA, USA, No. K 3469) until the requested staining appeared. This took up to 6 min for SCCs, 6 min for BCCs, 8 min for NCN and 5 min for MMs. The reaction was stopped with distillated water, and positive controls were counterstained with Mayer’s Haemalm (Roth, Karlsruhe, Germany, No. T865.3). The slides were scanned with PreciPoint M8, and the digital images were edited with ViewPoint online (PreciPoint, Freising, Bavaria, Germany).

### 2.2. Rating

Dermatopathologists assessed the staining of the sections visually. Sections were labelled as ++ for strong positive reactions with >80% of cells being positive and/or when staining intensity was high, + for 20–80% of cells demonstrating a weak positive/partial positive reaction, and − for <20% of cells displaying weak staining (a negative reaction). The epidermis was used as a reference structure in scoring. Tumors with a decrease in expression towards deeper tissue layers were considered weak positive. The same rating was applied for inconsistent staining throughout the tumor.

### 2.3. Statistics

First, all rating results for all entities were compared using Kruskal–Wallis tests. For NCN and MMs, epidermal and dermal portions were separately used for testing. Pairwise comparisons were made via Bonferroni tests. Secondly, pairwise comparisons of BCCs vs. SCCs and of MMs vs. NCN were made for each protein using a Mann–Whitney U test, and the results are given as exact significance (shown as 2*(1-tailed significance), not corrected for ties, for BCCs vs. SCCs and epidermal portions of NCN/MMs) or asymptotic significance (2-tailed, for dermal portions of NCN/MMs).

## 3. Results

Initially, we selected 25 tumors for each entity. Unfortunately, there was not enough tissue left on some of the selected paraffin blocks to perform all four stainings. Furthermore, staining did not work properly (e.g., over-staining) on some of the samples or did not show tumor cell nests on the sliced tissue, and therefore could not be used for evaluation. In the end, we were left with varying numbers for each combination of tumor and staining.

The mean age ± standard deviation was: SCCs: 85.43 ± 12.29 years (70% male and 30% female), BCCs: 81.19 ± 14.60 years (67% male and 33% female); NCN: 48.93 ± 16.86 years (53% male and 47% female); MMs: 66.48 ± 18.75 years (51% male and 49% female).

Figure 1 (SCC), Figure 2 (BCC), Figure 3 (NCN) and Figure 4 (MM) show representative IHC staining results, and the other samples are depicted in Supplementary Figures S1–S16. An overview of staining results/scores is shown in Figure 5. Positive/negative controls are included in the Supplementary Information (Supplementary Figures S17–S20). The scoring results for each tumor can be found in the Supplementary Information (for each protein in Supplementary Tables S1–S4, for each tumor in Supplementary Tables S5–S8).

Overall, NCN, MMs and SCCs appeared to be predominantly weak or strongly positive for GPR31, GPR151, TASK1 and TASK3, whereas BCCs showed no expression of proton-sensing receptors or ion channels in 50% of cases. Furthermore, we observed a decrease in the expression of proton-sensitive proteins in deeper tissue sections of some NCN and MMs.

Tumors with a decrease in expression towards deeper tissue layers were considered as being weakly positive. The same rating was applied for inconsistent staining throughout the tumor.

### 3.1. GPR31

**Figure 1:**
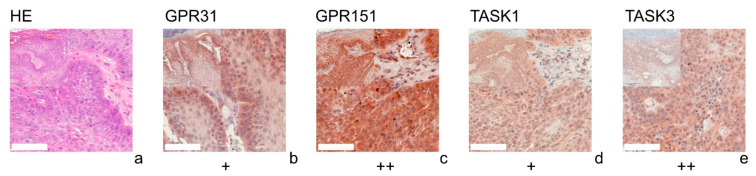
Immunohistochemistry of SCCs. Immunohistochemical staining for GPR31, GPR151, TASK1, and TASK3 in SCC tissue. (**a**) H&E staining, (**b**–**e**) Immunohistochemical staining. This SCC (patient 10 = slide number 8931) shows strong expression of GPR151 and TASK3. The tumor cells show a weak, positive expression of GPR31 and TASK1. For more stainings of other SCCs, see Supplementary Figures S1, S5, S9 and S13. Scale bars represent 200 μm. Overview inserts are two times lower in magnification.

**Figure 2:**
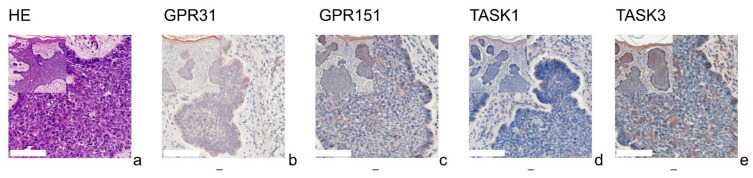
Immunohistochemistry of BCCs. Immunohistochemical staining for GPR31, GPR151, TASK1, and TASK3 in BCC tissue. (**a**) H&E staining, (**b**–**e**) immunohistochemical staining. This BCC (patient 2 = slide number 27) shows very low expression of GPR31, GPR151, TASK1 and TASK3. The epidermis serves as a reference with relatively uniform expression. For more stainings of other BCCs, see Supplementary Figures S2, S6, S10 and S14. Scale bars represent 200 μm. Overview inserts are two times lower in magnification.

**Figure 3:**
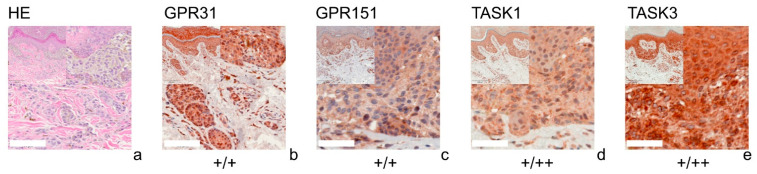
Immunohistochemistry of NCN. Immunohistochemical staining for GPR31, GPR151, TASK1 and TASK3 in NCN. (**a**) H&E staining, (**b**–**e**) immunohistochemical staining. This NCN (patient 3 = slide number 1256) shows weak positive expression of GPR31/GPR151. The tumor cells show a weak positive expression of TASK1 and TASK3 in epidermal parts of the tissue and strong positive expression in dermal portions. For more stainings of other NCN, see Supplementary Figures S3, S7, S11 and S15. Scale bars represent 200 μm. Overview inserts are two times lower in magnification.

**Figure 4:**
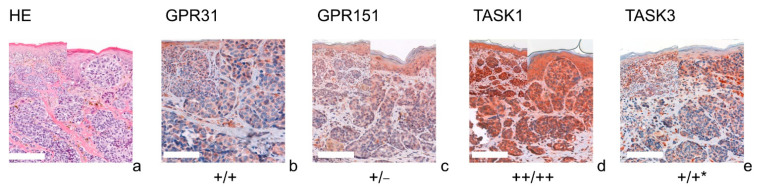
Immunohistochemistry of MMs. Immunohistochemical staining for GPR31, GPR151, TASK1 and TASK3 in MM. (**a**) H&E staining, (**b**–**e**) immunohistochemical staining. This MM (patient 13 = slide number 10968) shows strong expression of TASK1 in both epidermal and dermal parts of the tissue. GPR151 and TASK3 stainings show a weak, positive expression in epidermal tumor nests and negative (GPR151), respectively decreasing expression (TASK3) in dermal tumor nests. This is a typical example for the decreasing expression of the G-proteins/KCNKs in deeper tissue sections (marked with *). For more stainings of other MMs, see Supplementary Figures S4, S8, S12 and S16. Scale bars represent 200 μm. Overview inserts are two times lower in magnification.

All of the SCCs showed a weakly positive expression of GPR31 (Figure 1b, Supplementary Figure S1), whereas half of the BCC samples showed a weak expression (Figure 2b), and the other half were negative for GPR31 (Supplementary Figure S2). NCN showed a relatively homogeneous result with mainly weakly positive staining, except for 1/14 demonstrating strong positive and 1/14 displaying negative stainings in dermal regions, respectively (Figure 3b, Supplementary Figure S3). While only about 4/28 of the epidermal and dermal sections of MM appeared negative for GPR31 (Supplementary Figure S4), the main part of MM showed weak positive expression in both the epidermal (26/28) and dermal (20/28) portions (Figure 4b). The remaining 1/28 epidermal portions and 7/28 dermal portions showed strong GPR31 expression. GPR31 expression was significantly lower in BCCs as compared to SCCs as well as between BCCs and NCN/MMs (all *p* < 0.01 in Kruskal–Wallis and post hoc Bonferroni tests, *p* = 0.009 for BCCs vs. SCCs in Mann–Whitney U tests, details in Supplementary Tables S9 and S10). No significant differences in expression were found between NCN and MMs.

### 3.2. GPR151

In SCCs, GPR151 expression was weakly positive in 10/17, strongly positive in 6/17 and negative in only 1/17 of the cases (Figure 1c, Supplementary Figure S5). In comparison, BCCs appeared to be negative in 9/19 of the tissue samples, weakly positive in 8/19 and strongly positive in 2/19 of samples (Figure 2b and Supplementary Figure S6). Compound NCN exhibited strong positive expression of GPR151 in 2/18 of the epidermal tissue sections, while none of the dermal sections were strongly positive. In 12/18 NCN, we found a weakly positive expression of GPR151 in both epidermis and dermis. Four out of 18 of the epidermal and 6/18 of the dermal sections showed no expression of GPR151 (Figure 3c, Supplementary Figure S7). Only 1/23 of the epidermal parts of MM exhibited strong positive staining (none of dermal sections), while 16/23 of the epidermal and 14/23 of the dermal portions showed weak positive expression of GPR151. Six out of 23 epidermal and 9/23 dermal portions were negative for GPR151 (Figure 4c, Supplementary Figure S8). Overall, melanocytic tumors appear to lose this marker with increasing depth. GPR151 expression was significantly lower in BCCs as compared to SCCs (*p* < 0.01 in Kruskal–Wallis and post hoc Bonferroni tests, *p* = 0.009 for BCCs vs. SCCs in Mann–Whitney U tests, details in Supplementary Tables S9 and S10). Furthermore, SCC showed more frequent positive expression than MM (*p* < 0.01 Kruskal–Wallis and post hoc Bonferroni, details in Supplementary Tables S9 and S10, both epidermal and dermal). No significant differences in expression were found between NCN and MM.

### 3.3. TASK1

Only 1/16 SCCs showed a negative staining, while 13/16 showed a weakly positive and 2/16 showed a strong expression of TASK1 (Figure 1d, Supplementary Figure S9). Eight out of 20 of the BCC tissue samples showed no expression of TASK1 and 12/20 appeared to be weakly positive (Figure 2d, Supplementary Figure S10). NCN were weakly positive in 15/19 of the epidermal portions and in 14/19 of the dermal portions, negative in 4/19 of the epidermal and dermal tissue areas and strongly positive in 1/19 of dermal portions (Figure 3d, Supplementary Figure S11). Two out of 21 of the MMs showed a strong expression of TASK1 in dermal and epidermal portions. Weakly positive staining was seen in 9/21 epidermal and 12/21 dermal tissue sections. Negative staining results were found in 10/21 epidermal and 7/21 dermal portions of MMs (Figure 4d, Supplementary Figure S12). Overall, TASK1 seems to be more expressed in deeper sections of the tissue. TASK1 expression was significantly lower in BCCs as compared to SCCs (*p* < 0.01 Kruskal–Wallis and post hoc Bonferroni tests, *p* = 0.029 in Mann–Whitney U tests, details in Supplementary Tables S9 and S10). No significant differences in expression were found between NCN and MM.

### 3.4. TASK3

SCCs showed strong expression in 6/17 and weak expression in 10/17 of tissue samples. Only 1/17 was negative for TASK3 (Figure 1e, Supplementary Figure S13). Ten out of 18 of BCC showed a weak expression of TASK3, whereas 7/18 showed no expression and the remaining 1/18 showed a strong expression (Figure 2e, Supplementary Figure S14). In epidermal parts, 6/17 of NCN samples were strongly positive, 7/17 were weakly positive and 4/17 were negative for TASK3. Furthermore, dermal sections appeared to be weakly positive in 9/17 of NCN. The remaining 8/17 were evenly distributed between negative and strongly positive staining results (Figure 3e, Supplementary Figure S15). MM showed strongly positive staining in 4/21 epidermal and 5/21 dermal portions. Weakly positive staining was seen in 11/21 epidermal and 8/21 dermal portions. Negative results were observed in 5/21 epidermal and 7/21 dermal MM portions (Figure 4e, Supplementary Figure S16). TASK3 expression was significantly lower in BCCs as compared to SCCs (*p* < 0.01 Kruskal–Wallis and post hoc Bonferroni tests, *p* = 0.011 in Mann–Whitney U tests, details in Supplementary Tables S9 and S10). No significant differences in expression were found between NCN and MMs.

## 4. Discussion

We examined the expression profiles of GPR31, GPR151, TASK1 and TASK3 in the most common types of skin cancer. We found mixed results and inhomogeneous expression profiles of the different receptors even in the same tumor type, which might be explained by different patients with diverse genetics, unequal growth patterns/tumor thickness and opposing functions of receptors in the human body. There is also a high probability of channel/receptor interplay between pH-sensitive GPCRs and TASKs [30,31]. One of the most striking results found was the lower frequency of GPR31/151 and TASK1/3 expression in BCC when compared to SCC.

There is no difference in the RNA expression levels of the investigated proteins according to studies analyzed at NCBI Geo (Supplementary Table S11). However, protein expression was obviously altered between BCCs and SCCs, as seen in our immunohistochemistry stainings. BCCs seem to lose the pH-sensitive proteins investigated in this study. Studies on cBioportal were analyzed for mutation frequencies of GPR31, GPR151, KCNK3, and KCNK9 in the investigated tumor entities. For non-melanoma skin cancer, SCCs showed a markedly higher mutation frequency than BCCs (Supplementary Figure S21). However, protein expression was found to be reduced in BCCs as compared to SCCs, which could be due to post-transcriptional processes. For MMs, up to 8% mutation frequency was found for GPR31/151 and KCNK3. However, more than 30% mutations were found in one study on KCNK9 (Supplementary Figure S22). Interestingly, a large portion of these were classified as amplification. Further studies are needed to address the role of KCNK9/TASK3 in MMs’ progression from nevi.

### 4.1. GPR31/GPR151

GPR31 is evenly expressed in SCCs, MMs and in NCN in both epidermal and dermal portions, but NCN showed more negative staining results than MMs. In contrast to SCCs (100% +), BCCs were negative in ~50% of the samples, which is an interesting immunohistochemical feature to distinguish both entities. Even though there is little information on the expression of GPR31 in melanomas and NMSC, the role in cancer progression has been proven in other tissues. GPR31 expression is significantly upregulated in colorectal cancer tissues [16]. High GPR31 expression indicates poor prognosis of colorectal cancer. Furthermore, the expression of 12-HETER1 positively correlates with the grades of prostate cancer, and knockdown of GPR31 in cancer cells inhibited HCC recurrence in NAFLD [15,32,33]. Taking these considerations and our results into account together, GPR31 might serve as a potential diagnostic target, but more functional studies are required to fully understand the role of GPR31 in skin tumor progression.

Among all investigated tumors, BCC showed the lowest expression rates of GPR151. Contrarily, nearly all SCCs showed positive expression of GPR151. In MMs and NCN, we found inhomogeneous results concerning the expression of GPR151 in epidermal and dermal tissue portions.

Another interesting observation was the weak or strong positive staining of the lymphocytes. As Morita et al. found, in CX3CR1+ immune cells, GPR31 is activated in the intestinal lumen, leading to their dendrite protrusion and enhancing intestinal immune responses [34], while Lämmermann et al. summarized GPCRs and especially the proton-sensitive GPR31 and GPR151, which are commonly found in immune cells [35].

### 4.2. TASK1/TASK3

All investigated tumors, except for BCCs, showed frequent expression of TASK3. As mentioned before, TASK3 is responsible for maintaining the resting membrane potential of the cell. Besides TASK3, numerous K^+^ channels have been reported to be differentially expressed in human cancer and regulate different aspects of tumorigenicity [28,36]. The mechanism responsible for this connection is the hyperpolarization caused by the opening of TASK3 due to acidification, which then promotes cell cycle progression through G_1_–S. We can say that TASK1/3 are widely expressed in the more aggressive common skin tumors SCC and MM, but are rarely found to be highly expressed in BCC. As Sun et al. found that the inhibition of TASK3 via Y4 reduced the proliferative index and decreased the tumor burden in vivo, TASK3 could also be a promising therapeutic target in cancer [37].

TASK1 shows quite similar expression patterns to TASK3, except for MMs, where it is expressed less frequently. In MMs, we found mixed results for TASK1 expression, and in the dermal portion of NCN, no/low expression of TASK1 was predominant. Antigny et al. found that TASK1 inhibition promoted increased proliferation, vasoconstriction and inflammation [38]. Based on the clearly positive staining of lymphocytes for TASK1/3, we can confirm that these K⁺- channels are expressed in lymphocytes and therefore play a significant role in the human immune response, which makes them a potential drug target for T cell-mediated autoimmune diseases such as multiple sclerosis [39].

## 5. Conclusions

Taking everything into account, our findings need to be verified by a larger sample size and by the study of different patient collectives (based on the hypothesis that the expression of GPCRs/TASKs varies between patients, type of cancer and micro- and macroenvironmental circumstances) to investigate the different roles of GPCRs/TASKs more precisely.

As this is the first study on the expression of these pH-sensitive proteins (GPR31/151, TASK1/3) in skin tumors in the literature, our approach is descriptive. However, one of the most striking results found was the lower frequency of GPR31/151 and TASK1/3 expression in BCC when compared to SCC and melanocytic tumors (NCN/MM). This makes the study of these pH-sensitive proteins a novel histological/diagnostic tool to distinguish these two entities (BCC/SCC). GPR31 is expressed in all SCC but only in half of the BCC.

In terms of methodology, automated evaluation of immunohistochemistry could be a potential approach for future studies [40]. As a further step, it would then be important to conduct clinical studies on whether these receptors/channels have a significant effect on the overall survival of MM patients. In a similar context, studies should focus on the expression of GPR31, GPR151, and TASK1/3 in metastases of SCCs and MMs. Further studies could focus on how different growth patterns or tumor stages correlate with the expression of the investigated proteins.

## Figures and Tables

**Figure 5:**
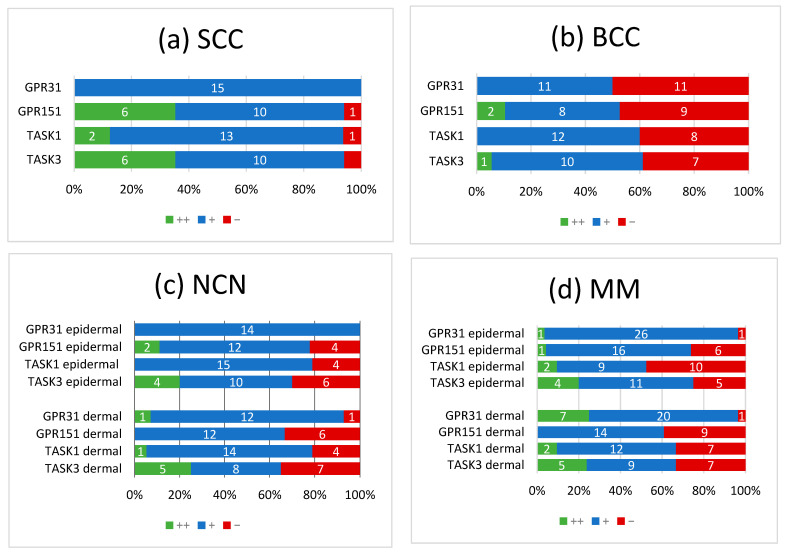
Summary of immunohistochemical scoring results for GPR31, GPR151, TASK1 and TASK3 on (**a**) SCCs, (**b**) BCCs, (**c**) NCN and (**d**) MMs. ++/green bar: strongly positive staining with >80% of cells positive and/or staining intensity is high; +/blue bar: 20–80% of cells show a weakly positive/partially positive reaction; −/red bar: <20% of cells with weak staining (=negative reaction). NCN and MM are subdivided into epidermal and dermal portions. Numbers in bars represent the occurrence of the particular score. For additional information on the individual scores, see Supplementary Tables S1–S4.

## Data Availability

Available upon reasonable request.

## Supplementary Materials

The following are available online at https://www.mdpi.com/article/10.3390/cells11010027/s1, Figures S1–S4: Immunohistochemistry for GPR31, Figures S5–S8: Immunohistochemistry for GPR151, Figures S9–S12: Immunohistochemistry for TASK1, Figures S13–S16: Immunohistochemistry for TASK3, Figures S17–S20: Positive and negative controls, Figures S21 and S22: Mutation frequencies of GPR31, GPR151, TASK1, TASK3 in non-melanoma skin cancer and melanoma., Table S1: Scoring for GPR31, Table S2: Scoring for GPR151, Table S3: Scoring for TASK1, Table S4: Scoring for TASK3, Tables S5–S8: Scoring results for each tumor entity, Tables S9 and S10: Statistical analysis test results, Table S11: NCBI Geo mRNA expression comparison results.
