# Supplementary material for: Expression of Proton-Sensitive GPR31, GPR151, TASK1 and TASK3 in Common Skin Tumors
Source: Cells. 2021 Dec 23;11(1):27. doi: 10.3390/cells11010027 (PMC8744809; doi:10.3390/cells11010027)
Supplement: Supplementary file 1 [file cells-11-00027-s001.zip › cells-1509301-supplementary.pdf]

SUPPLEMENTARY INFORMATION

Supplementary Figure S1: Immunohistochemistry for GPR31 of SCC.

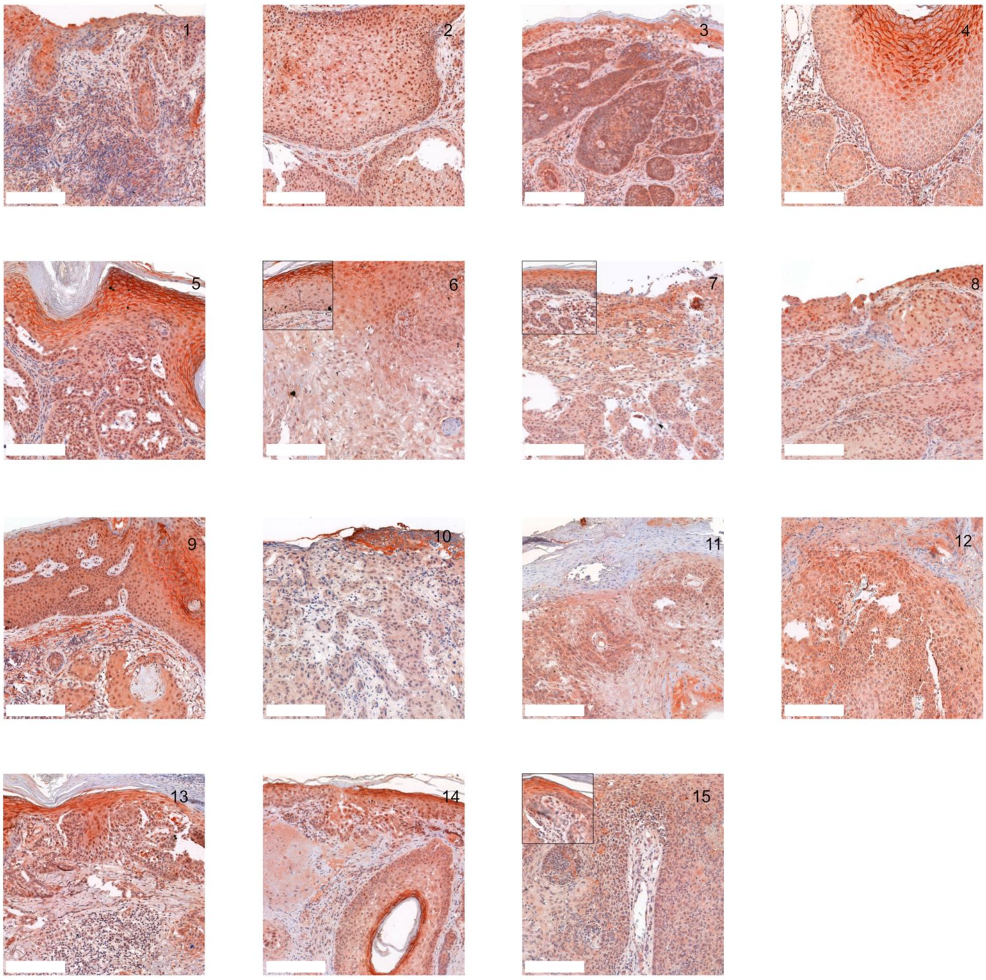

Supplementary Figure S1: Immunohistochemistry for GPR31 of SCC. Scale bars represent 200 µm. All of the SCC tissues showed weak positive expression.

Number 1 corresponds to slide number 9314, 2 = 12791, 3 = 9884, 4 = 11160, 5 = 11049, 6 = 16190, 7 = 9885, 8 = 10979, 9 = 7578, 10 = 8931, 11 = 8196, 12 = 2795, 13 = 9576, 14 = 7973, 15 = 8031

Supplementary Figure S2. Immunohistochemistry for GPR31 of BCC.

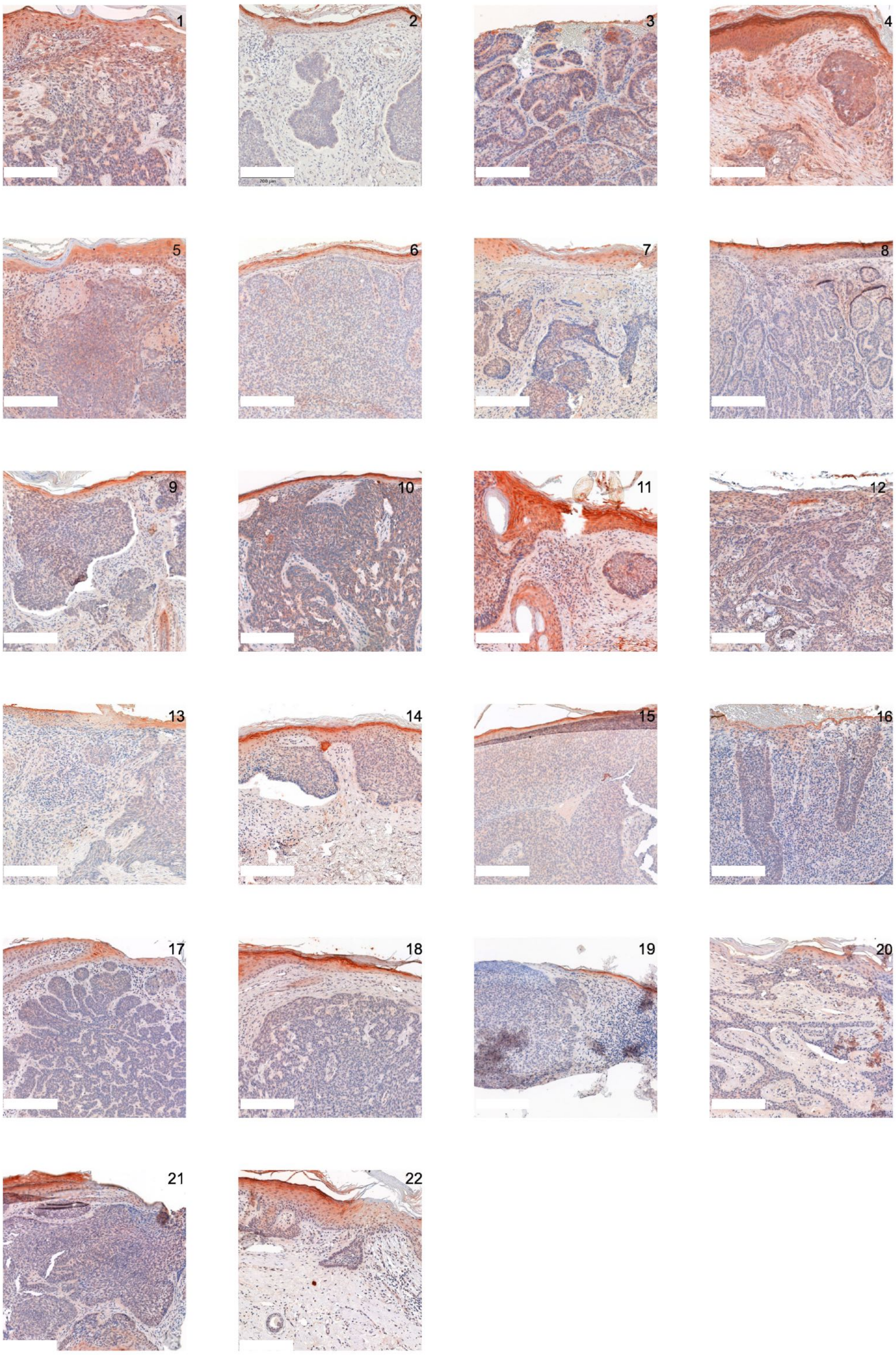

Supplementary Figure S2: Immunohistochemistry for GPR31 of BCC. Scale bars represent 200 µm. 50% of the BCC samples showed a weak expression and the other 50% were negative for GPR31.

1 = 410, 2 = 27, 3 = 409, 4 = 1589, 5 = 1677, 6 = 1393, 7 = 1587, 8 = 1473, 9 = 837, 10 = 1571, 11 = 1904, 12 = 2131, 13 = 1710, 14 = 1750, 15 = 1393, 16 = 816, 17 = 1048, 18 = 1518, 19 = 1732, 20 = 1714, 21 = 1858, 22 = 732

Supplementary Figure S3. Immunohistochemistry for GPR31 of NCN.

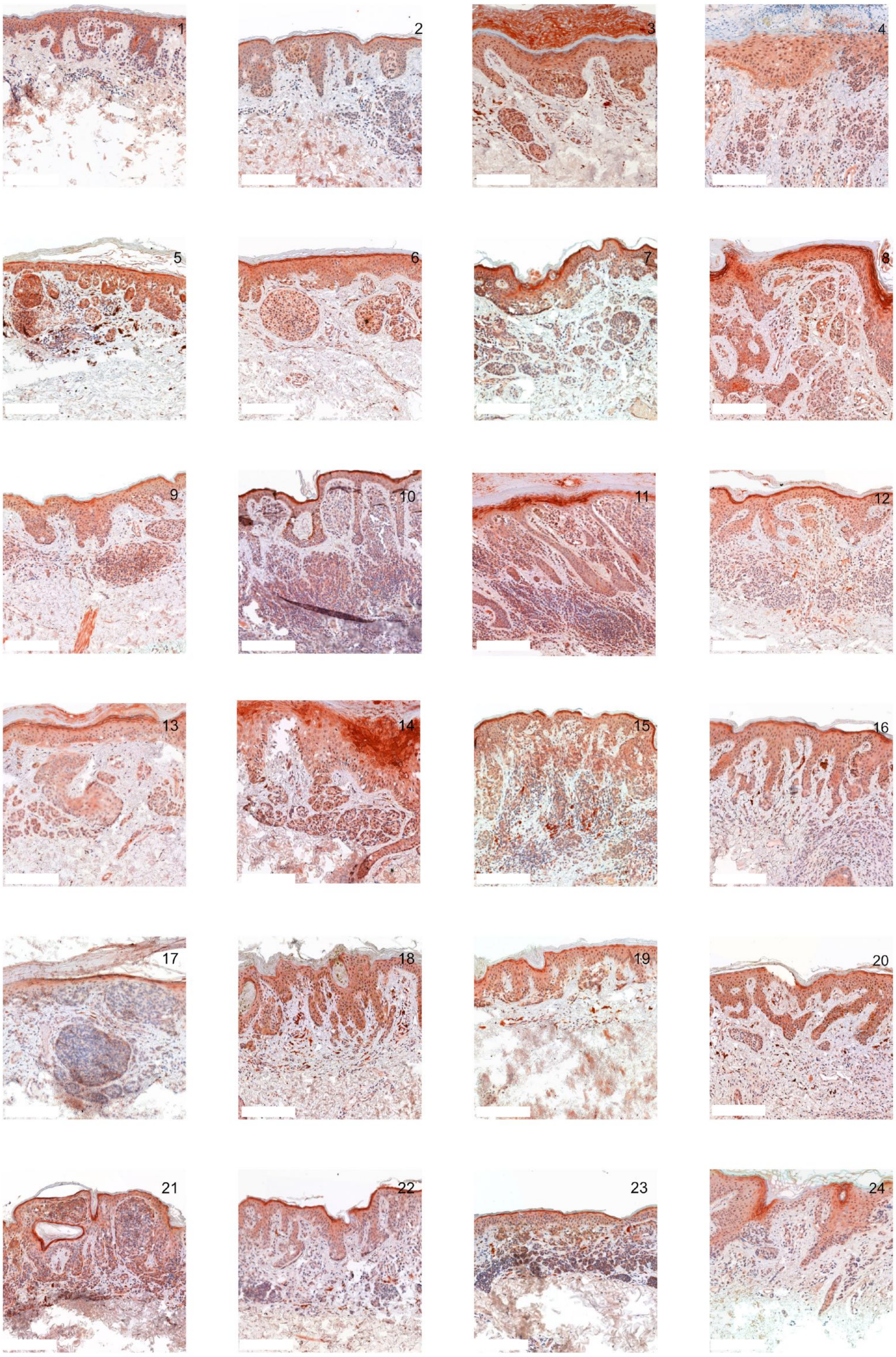

Supplementary Figure S3: Immunohistochemistry for GPR31 of NCN. Scale bars represent 200 µm. NCN showed a relatively homogeneous result with mainly weak positive staining except for 1/14 strong positive and 1/14 negative stainings in dermal regions, respectively.

1 = 254, 2 = 9285-09, 3 = 1256, 4 = 8134-09, 5 = 265, 6 = 30, 7 = 32, 8 = 34, [1)]9= 41, [1)]10 = 61, [1)]11 = 75, 12 = 101, [1)]13 = 105, 14 = 492, 15 = 114, [3)]16 = 116, [3)]17 = 117, [1)]18 = 121, 19 = 122, [1)]20 = 496, [1)]21 = 154, 22 = 156, 23 = 223, 24 = 244

[1] epidermal part is not expressed or not evaluable, [2] dermal part is not expressed or not evaluable, [3] not suitable for evaluation

Supplementary Figure S4. Immunohistochemistry for GPR31 of MM.

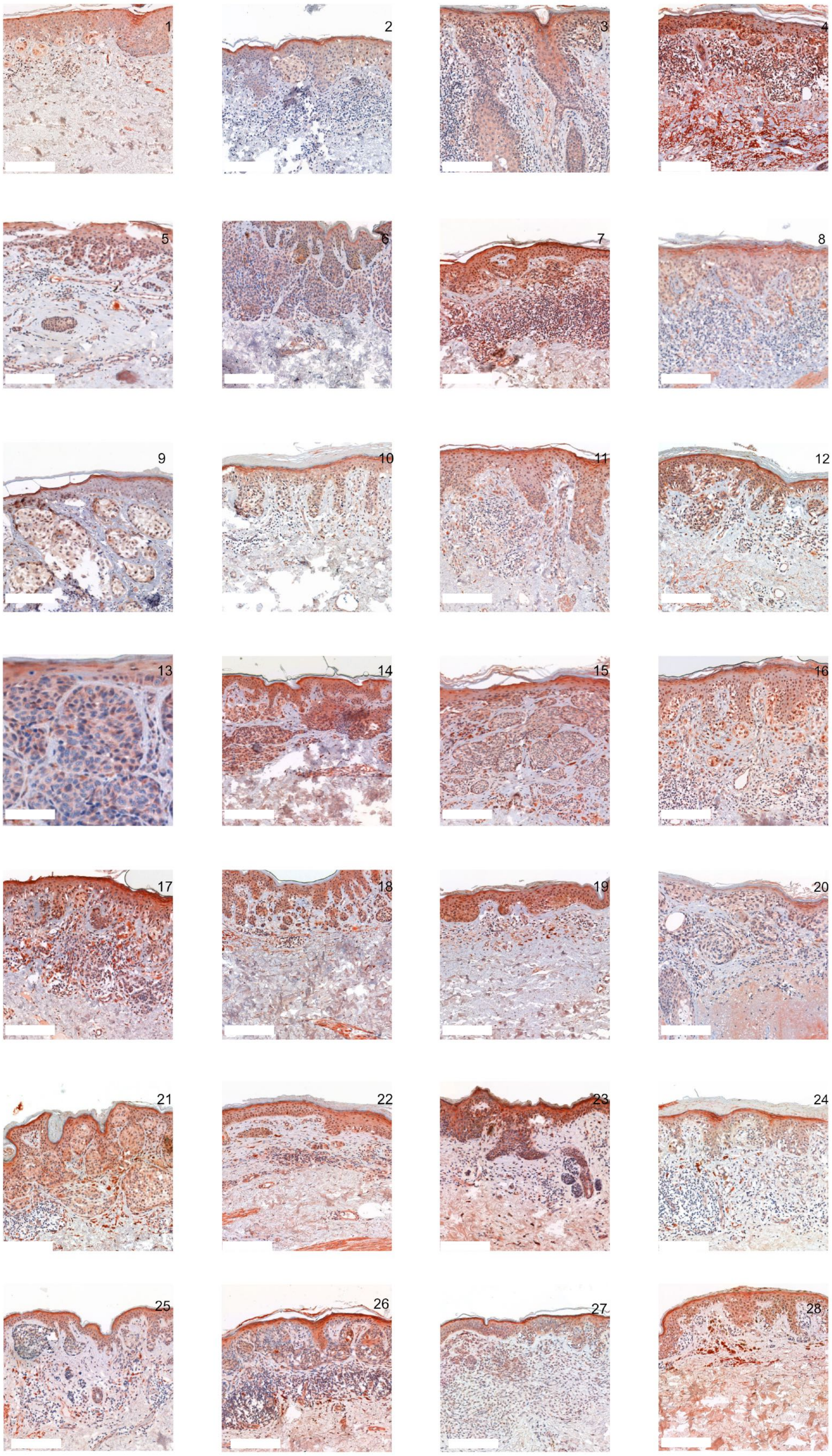

Supplementary Figure S4: Immunohistochemistry for GPR31 of MM. Scale bars represent 200 µm. 4/28 of the epidermal and dermal sections of MM appeared negative for GPR31, the main part of MM showed weak positive expression in both epidermal (26/28) and dermal (20/28) portions. The remaining 1/28 epi-dermal portions and 7/28 dermal portions showed strong GPR31 expression.

1 = 6926, 2 = 7439, 3 = 11094, 4 = 9207, 5 = 9346, 6 = 1345, 7 = 881, 8 = 9199, 9 = 8190, 10 = 8718, 11 = 7104, 12 = 4476, 13 = 10968, 14 = 11264, 15 = 11317, 16 = 3341, 17 = 7802, 18 = 11275, 19 = 3679, 20 = 7066, 21 = 812-11, 22 = 757-11, 23 = 1817-11, 24 = 1903-11, 25 = 2017-11, 26 = 2125-11, 27 = 2668-11, 28 = 3065-11

Supplementary Figure S5. Immunohistochemistry for GPR151 of SCC.

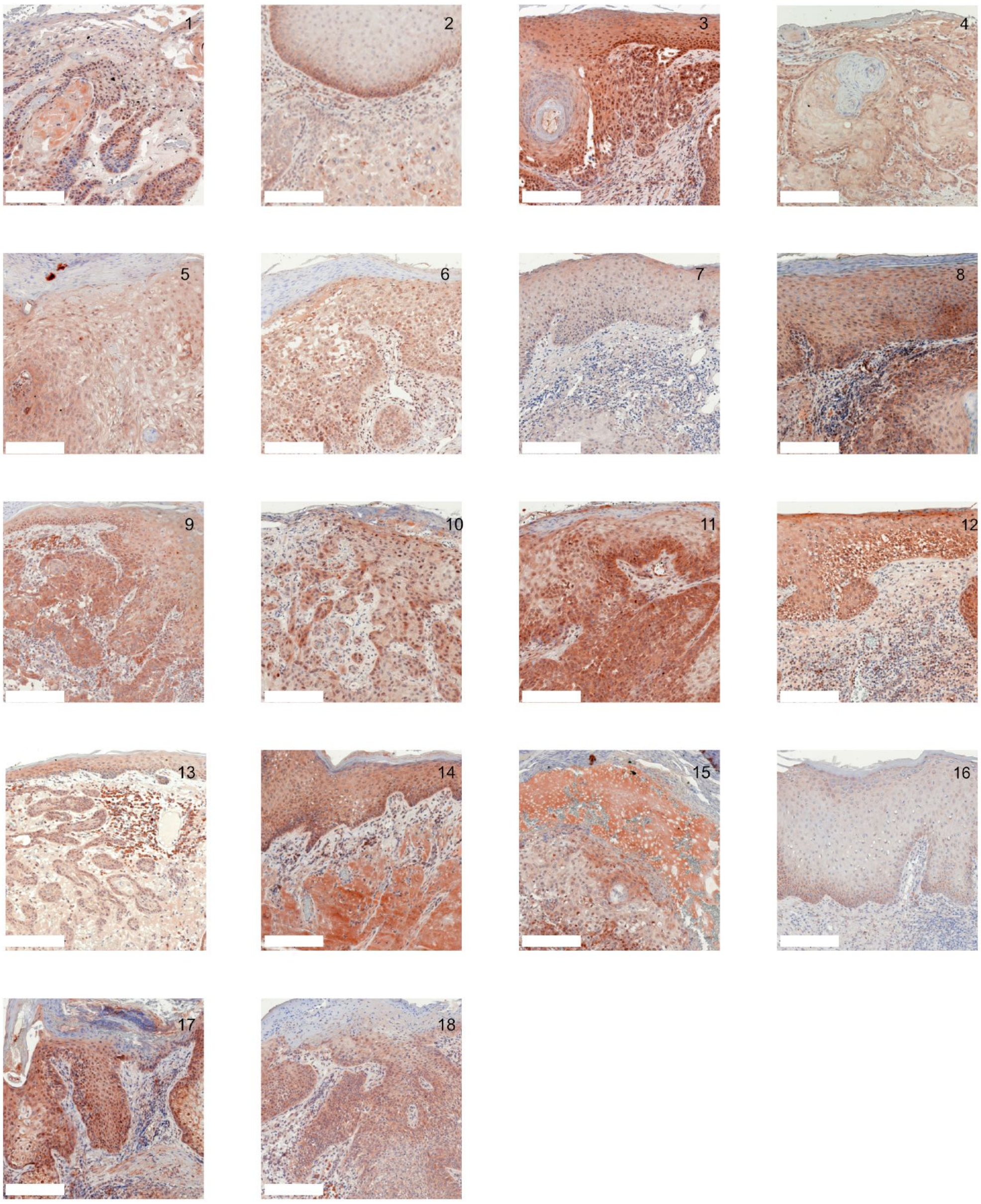

Supplementary Figure S5: Immunohistochemistry for GPR151 of SCC. Scale bars represent 200 µm. In SCC, GPR151 expression was weak positive in 10/17, strong positive in 6/17 and negative in only 1/17 of the cases.

1 = 1271, 2 = 10173, 3 = 11044, 4 = 1013, 5 = 11190, 6 = 1389, 7 = 2346, 8 = 6837, 9 = 7996, 10 = 8931, 11 = 9314, 12 = 9880, 13 = 9885, 14 = 12597, 15 = 7346, 16 = 2433, [3)]17 = 1279, 18 = 9576

[3)] not suitable for evaluation

Supplementary Figure S6: Immunohistochemistry for GPR151 of BCC.

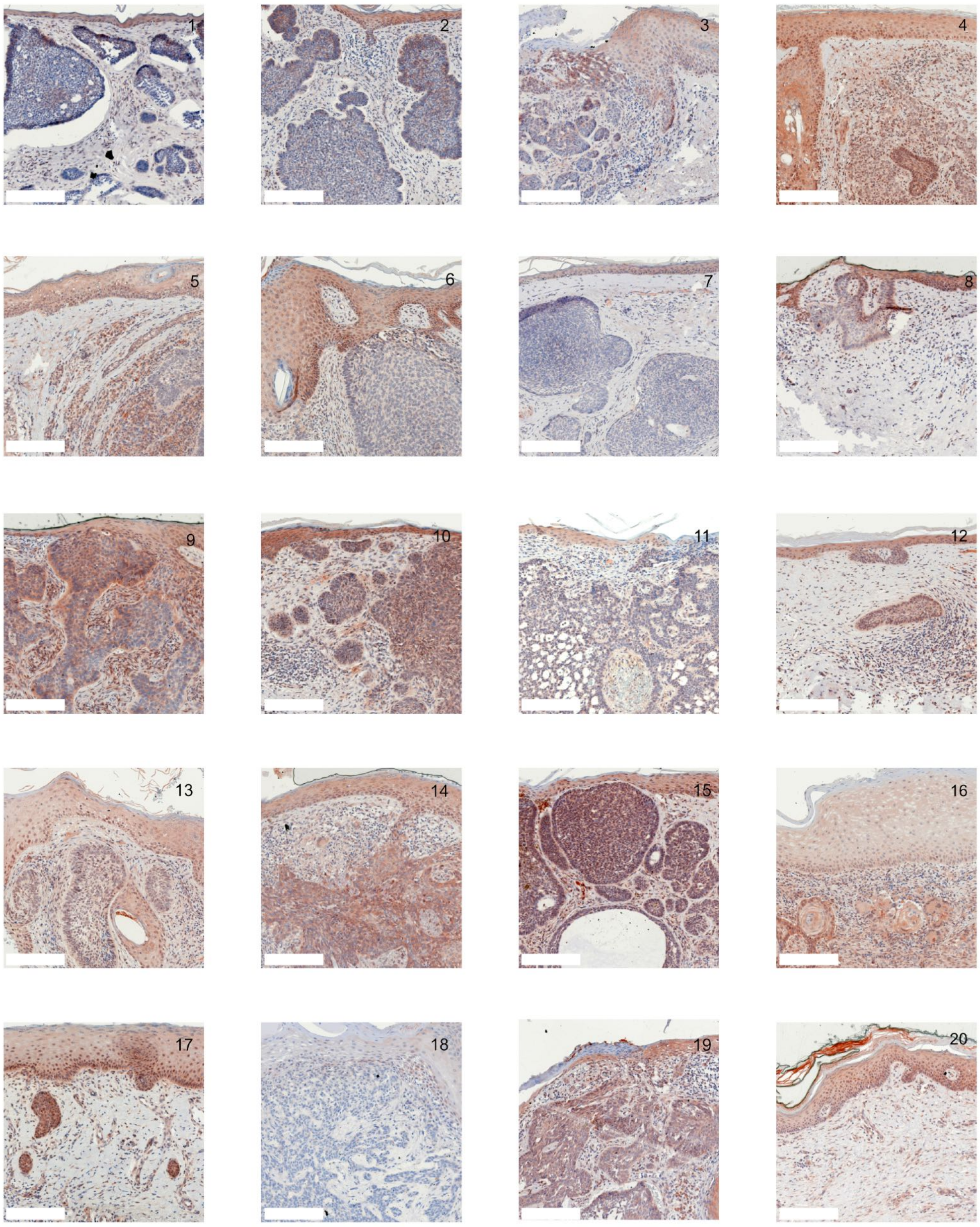

Supplementary Figure S6: Immunohistochemistry for GPR151 of BCC. Scale bars represent 200 µm. BCC appeared negative in 9/19 of the tissue samples, weak positive in 8/19 and strong positive in 2/19.

1 = 26, 2 = 27, 3 = 270, 4 = 796, 5 = 816, 6 = 845, 7 = 899, 8 = 911, 9 = 925, 10 = 1000, 11 = 1404, 12 = 1422, 13 = 1904, 14 = 2131, 15 = 999, 16 = 2752, 17 = 722, [3)]18 = 410, 19 = 877, [3)]20 = 1440, 21 = 265

[3)] not suitable for evaluation

Supplementary Figure S7. Immunohistochemistry for GPR151 of NCN.

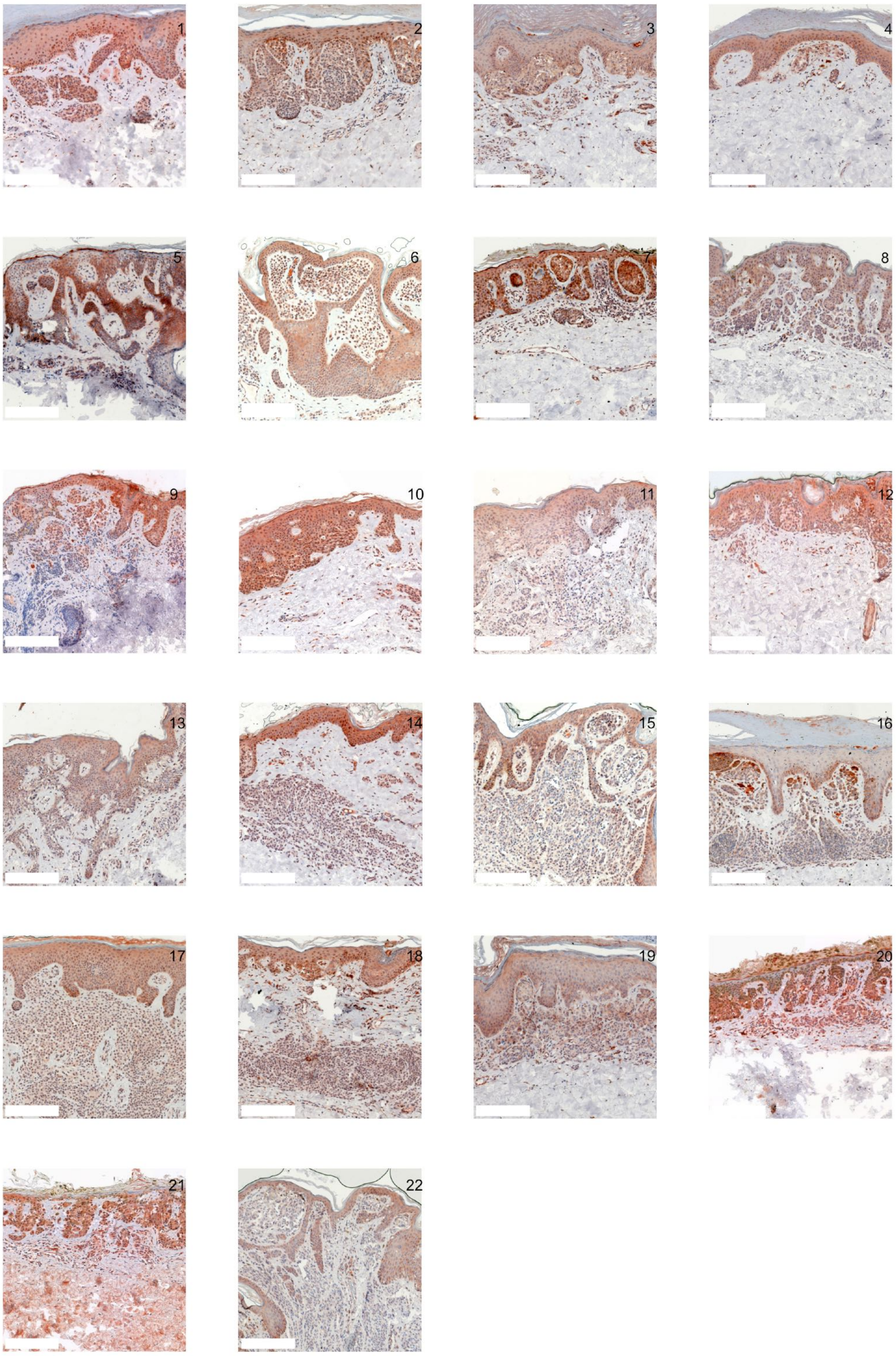

Supplementary Figure S7: Immunohistochemistry for GPR151 of NCN. Scale bars represent 200 µm. Compound NCN, exhibited strong positive expression of GPR151 in 2/18 of the epi-dermal tissue sections, while none of the dermal sections was strong positive. In 12/18 NCN, we found weak positive expression of GPR151 in both epidermis and dermis. 4/18 of epidermal and 6/18 of dermal sections showed no expression of GPR151.

1 = 986, 2 = 1054, 3 = 1256, 4 = 1580, 5 = 3795, [1)]6 = 26170, [1)]7 = 26480, 8 = 28814, 9 = 1496, 10 = 2369, 11 = 10092, 12 = 29215, 13 = 5950, 14 = 9285, 15 = 17735, 16 = 19116, 17 = 4251, [1)]18 = 1060, 19 = 27639, 20 = 27629, 21 = 18172

[1)] epidermal part is not expressed or not evaluable

Supplementary Figure S8. Immunohistochemistry for GPR151 of MM.

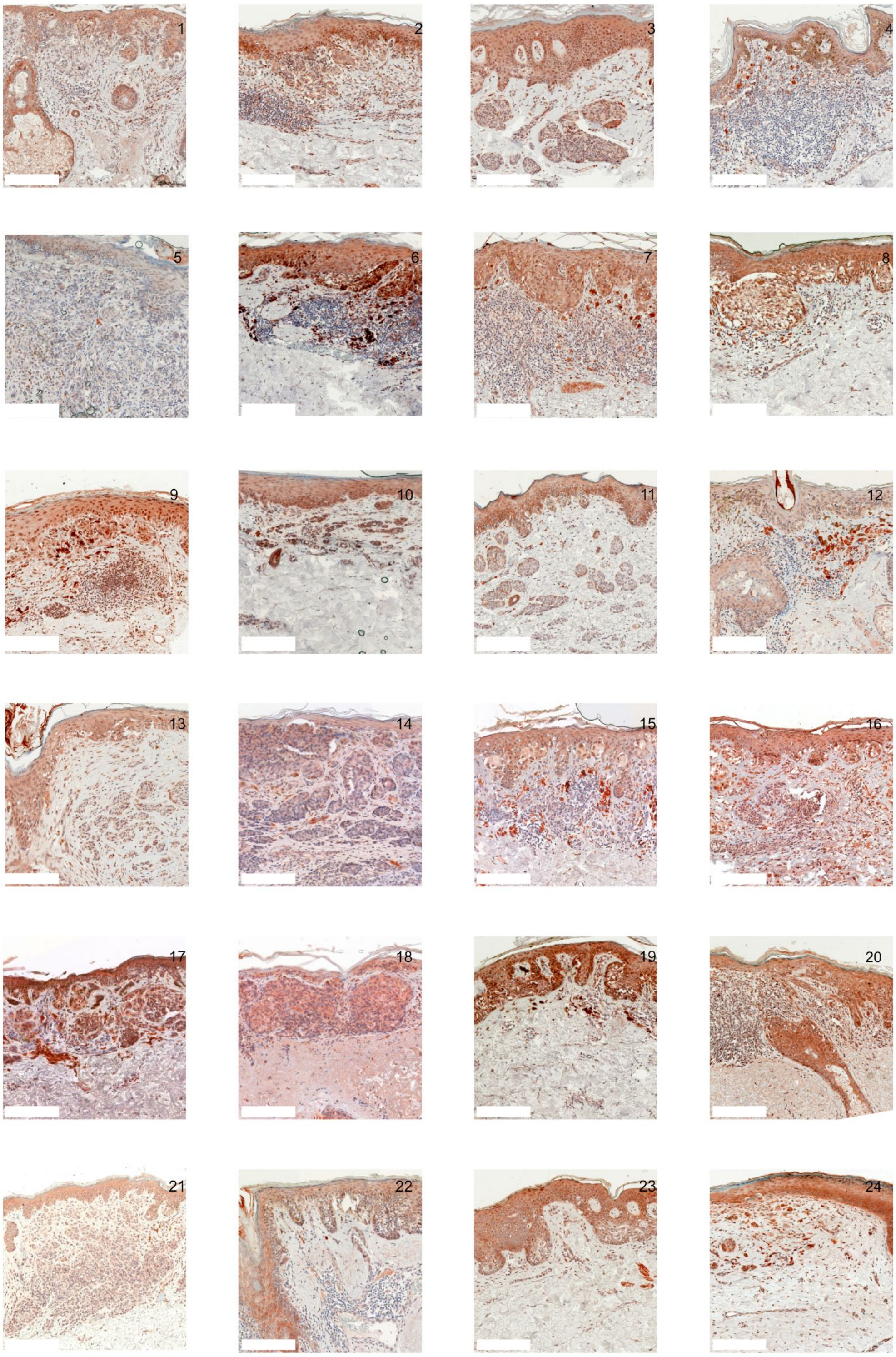

Supplementary Figure S8: Immunohistochemistry for GPR151 of MM. Scale bars represent 200 µm. Only 1/23 of the epidermal parts of MM exhibited strong positive staining (none of dermal sections), while 16/23 in epidermal and 14/23 in dermal portions showed weak positive expression of GPR151. 6/23 epidermal, respectively 9/23 of dermal portions were negative for GPR151.

1 = 730, 2 = 881, 3 = 1316, 4 = 2145, 5 = 5847, 6 = 7781, 7 = 9199, 8 = 9240, 9 = 9255, 10 = 9346, 11 = 9525, 12 = 10891, 13 = 10968, 14 = 7677, 15 = 7802, 16 = 8190, 17 = 9375, 18 = 7060, [3)]19 = 9256, 20 = 9207, 21 = 2239, 22 = 11094, 23 = 1447, 24 = 16846

[3)] not suitable for evaluation

Supplementary Figure S9. Immunohistochemistry for TASK1 of SCC.

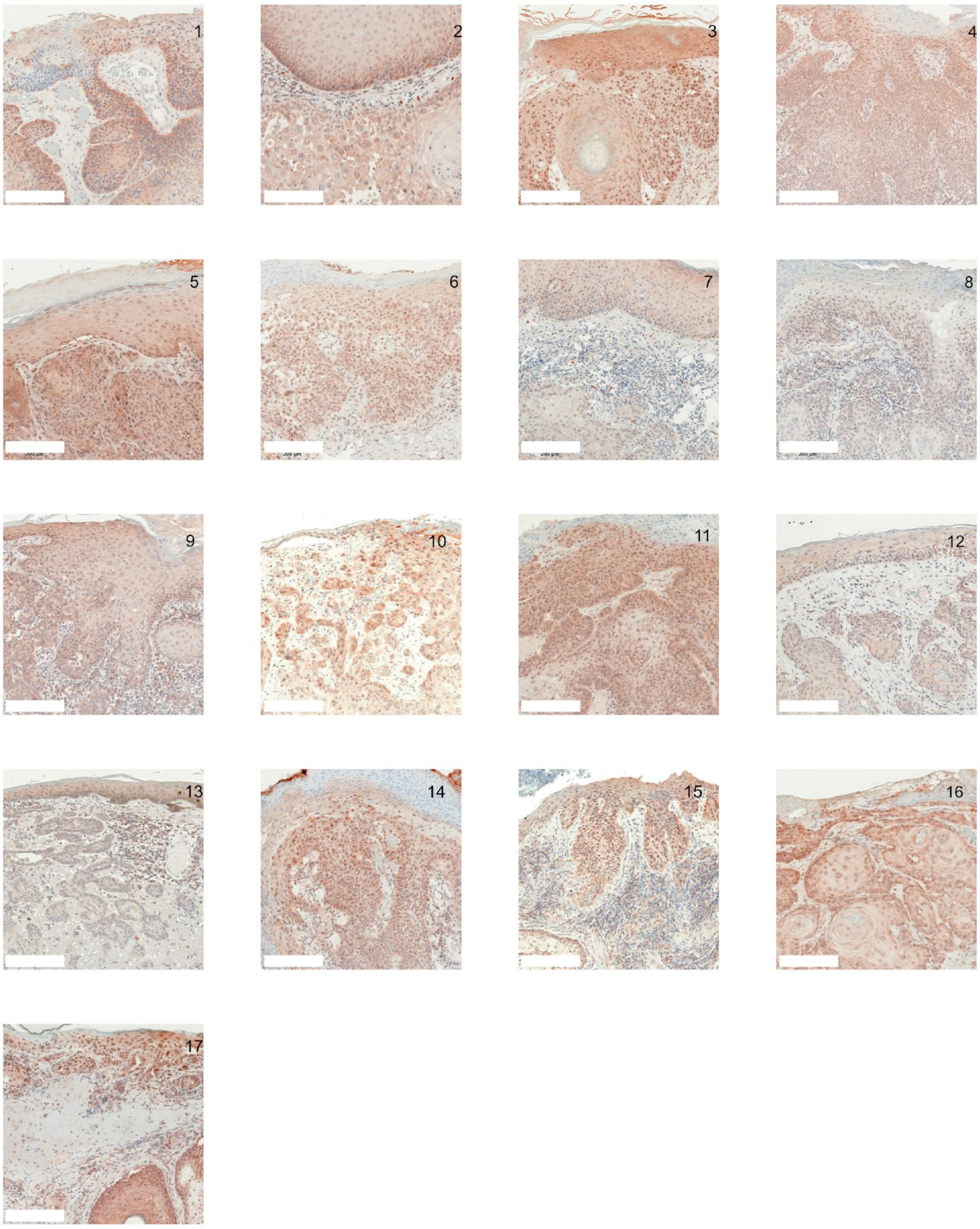

Supplementary Figure S9: Immunohistochemistry for TASK1 of SCC. Scale bars represent 200 µm. Only 1/16 SCC showed a negative staining, while 13/16 showed weak positive and 2/16 strong expression of TASK1.

1 = 1271, 2 = 10173, 3 = 11044, 4 = 1013, 5 = 11190, 6 = 1389, 7 = 2346, 8 = 6837, 9 = 7996, 10 = 8931, 11 = 9314, 12 = 9880, 13 = 9885, 14 = 12597, [3)]15 = 7346, 16 = 2433, [3)]17 = 1279, 18 = 9576

[3)] not suitable for evaluation

Supplementary Figure S10. Immunohistochemistry for TASK1 of BCC.

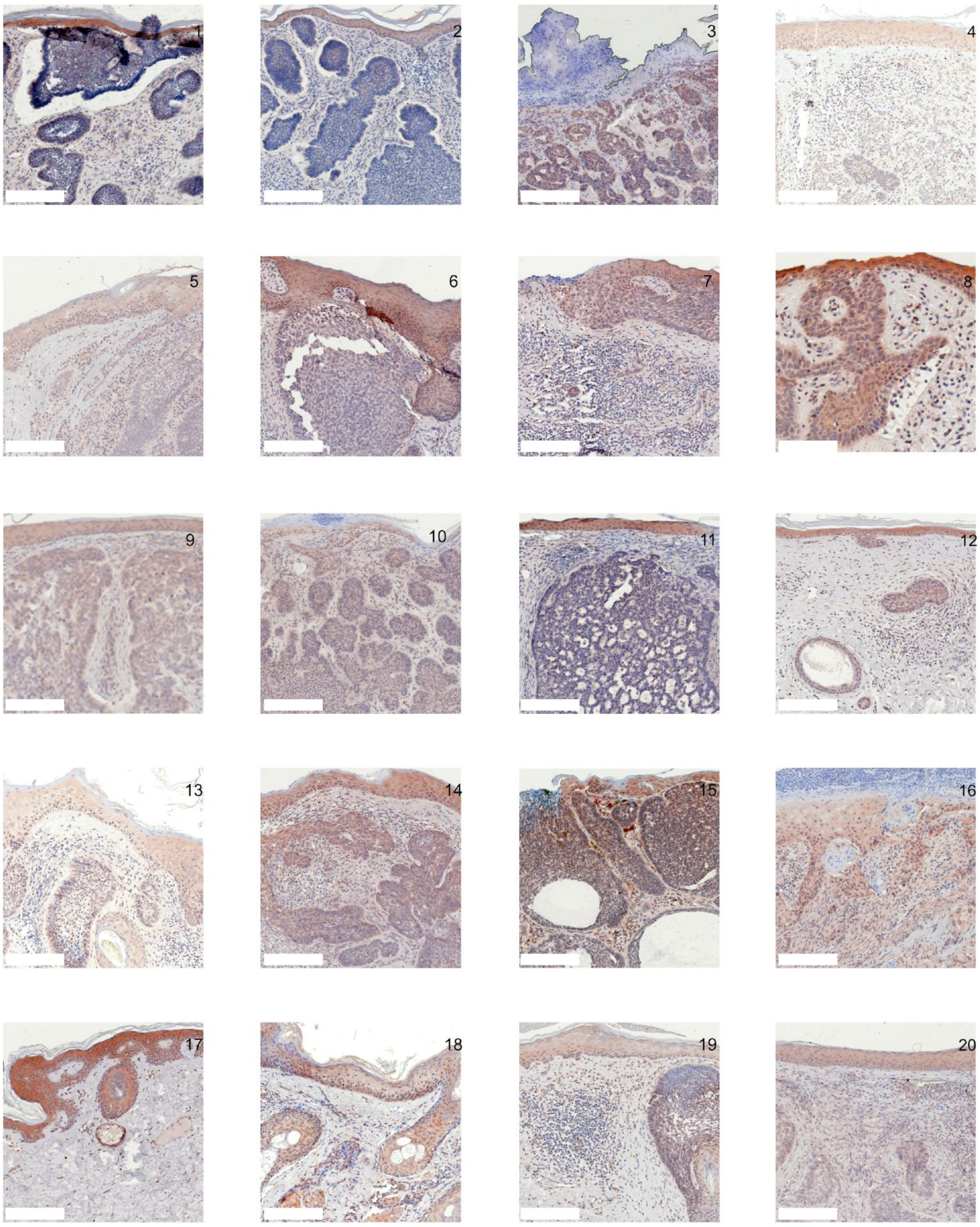

Supplementary Figure S10: Immunohistochemistry for TASK1 of BCC. Scale bars represent 200 µm. 8/20 of BCC tissue samples showed no expression of TASK1 and 12/20 appeared weak positive.

1 = 26, 2 = 27, 3 = 270, 4 = 796, 5 = 816, 6 = 845, 7 = 899, 8 = 911, 9 = 925, 10 = 1000, 11 = 1404, 12 = 1422, 13 = 1904, 14 = 2131, 15 = 999, [3)]16 = 2752, 17 = 1440, 18 = 410, 19 = 1438, 20 = 985,

[3)] not suitable for evaluation

Supplementary Figure S11. Immunohistochemistry for TASK1 of NCN.

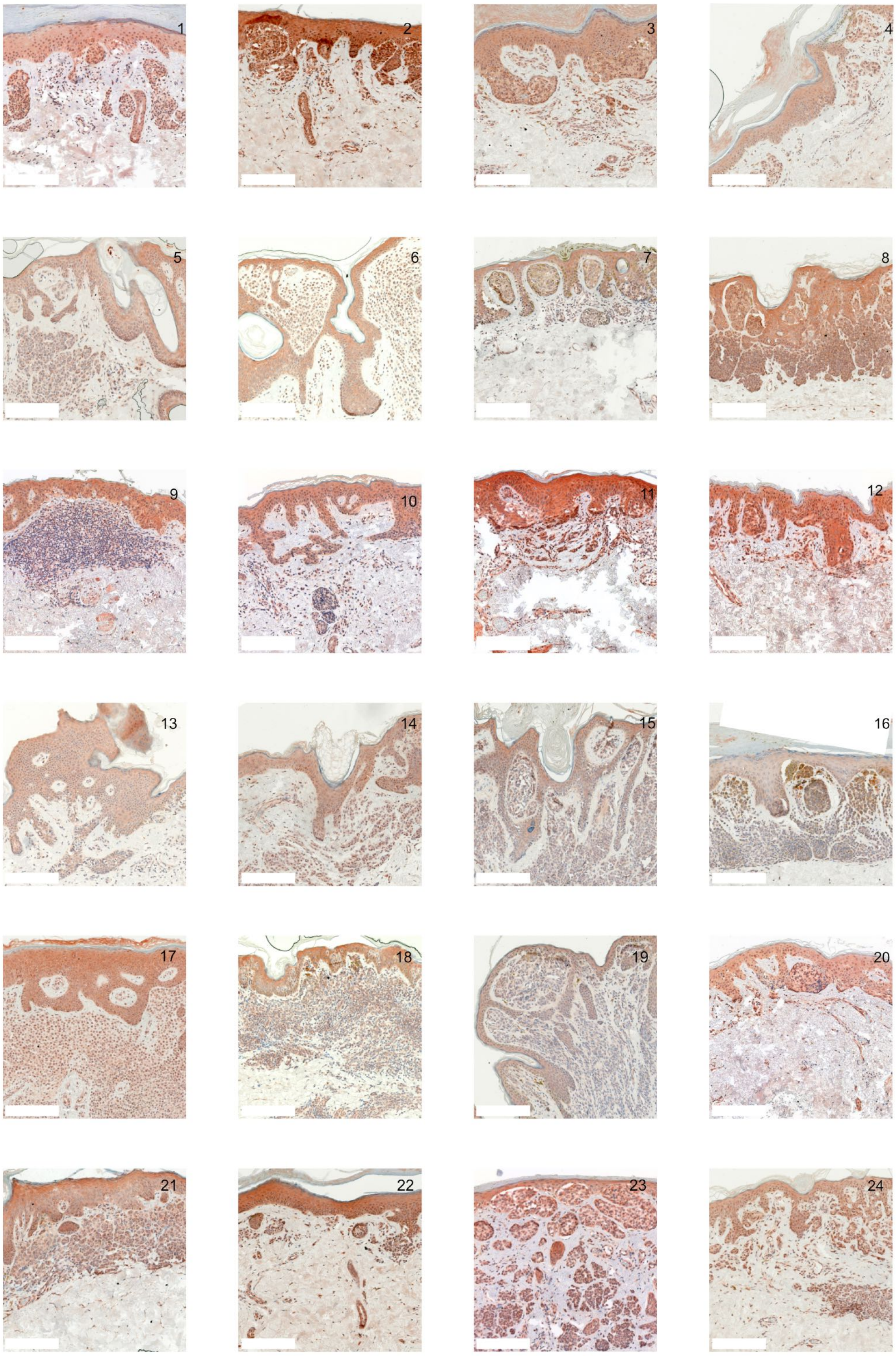

Supplementary Figure S11: Immunohistochemistry for TASK1 of NCN. Scale bars represent 200 µm. NCN were weak positive in in 15/1 of epidermal portions and in 14/19 of dermal portions, negative in 4/19 of epidermal and dermal tissue areas and strong positive in 1/19 of dermal portions.

1 = 986, 2 = 1054, 3 = 1256, 4 = 1580, 5 = 3795, [1)]6 = 26170, 7 = 26480, 8 = 28814, 9 = 1496, 10 = 2369, 11 = 10092, 12 = 29215, 13 = 5950, 14 = 9285, 15 = 17735, 16 = 19116, 17 = 4251, [1)]18 = 31892, [1)]19 = 18172, [2)]20 = 22545, 21 = 10076, 22 = 16427, 23 = 9625, [1)]24 = 106

[1)] epidermal part is not expressed or not evaluable

[2)] dermal part is not expressed or not evaluable

Supplementary Figure S12. Immunohistochemistry for TASK1 of MM.

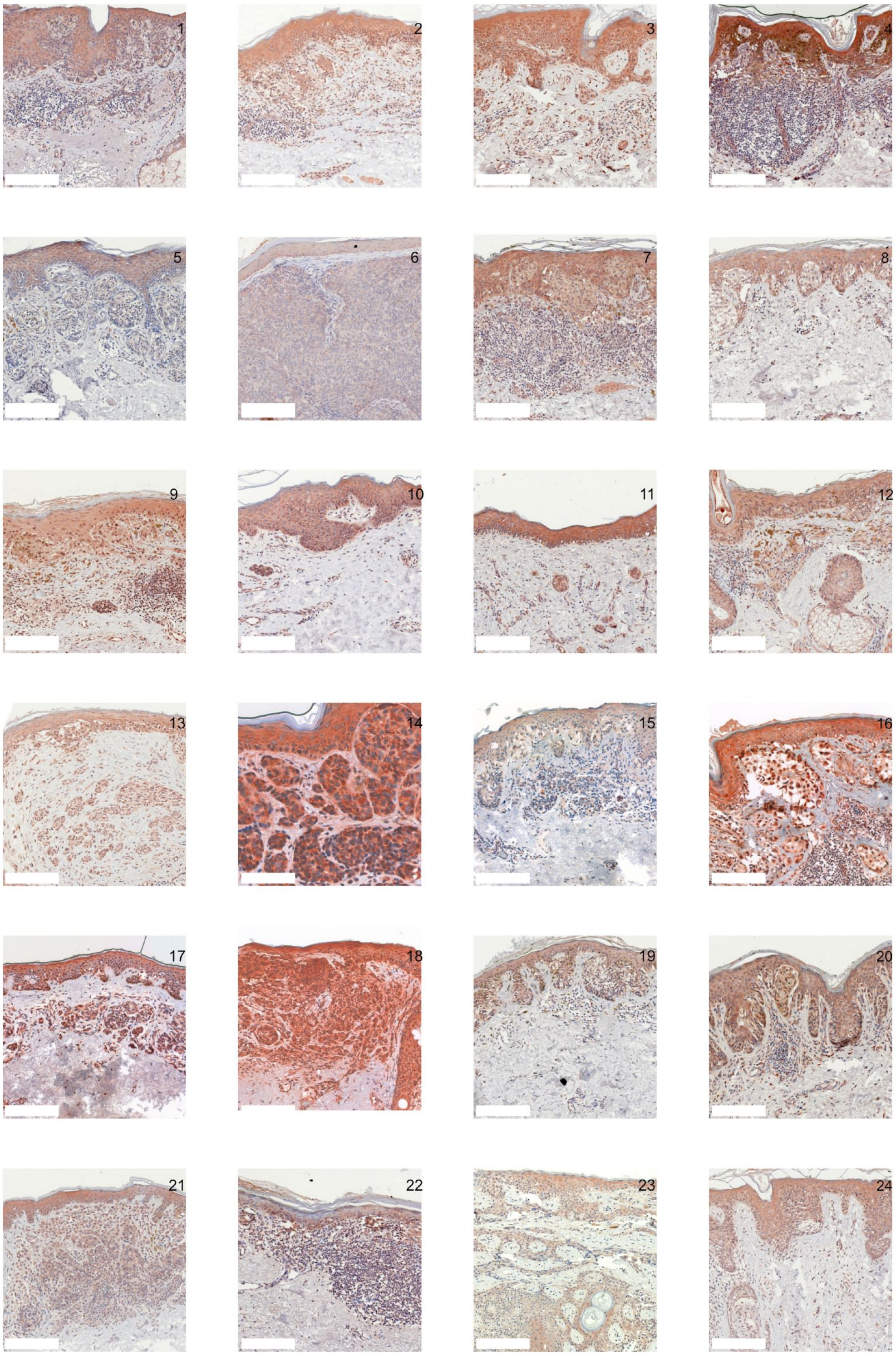

Supplementary Figure S12: Immunohistochemistry for TASK1 of MM. Scale bars represent 200 µm. 2/21 of MM showed strong expression of TASK1 in dermal and epidermal portions. Weak positive staining was seen in 9/21 of epidermal and 12/21 dermal tissue sections. Negative staining results were found in 10/21 epidermal and 7/21 dermal portions of MM.

1 = 730, 2 = 881, 3 = 1316, 4 = 2145, 5 = 5847, 6 = 7781, 7 = 9199, 8 = 9240, 9 = 9255, 10 = 9346, 11 = 9525, 12 = 10891, 13 = 10968, 14 = 7677, 15 = 7802, 16 = 8190, 17 = 9375, 18 = 7060, 19 = 9256, [1)]20 = 9089, [2)]21 = 2293, [3)]22 = 9207, [3)]23 = 1089, [1)]24 = 11094

[1)] epidermal part is not expressed or not evaluable

[2)] dermal part is not expressed or not evaluable

[3)] not suitable for evaluation

Supplementary Figure S13. Immunohistochemistry for TASK3 of SCC.

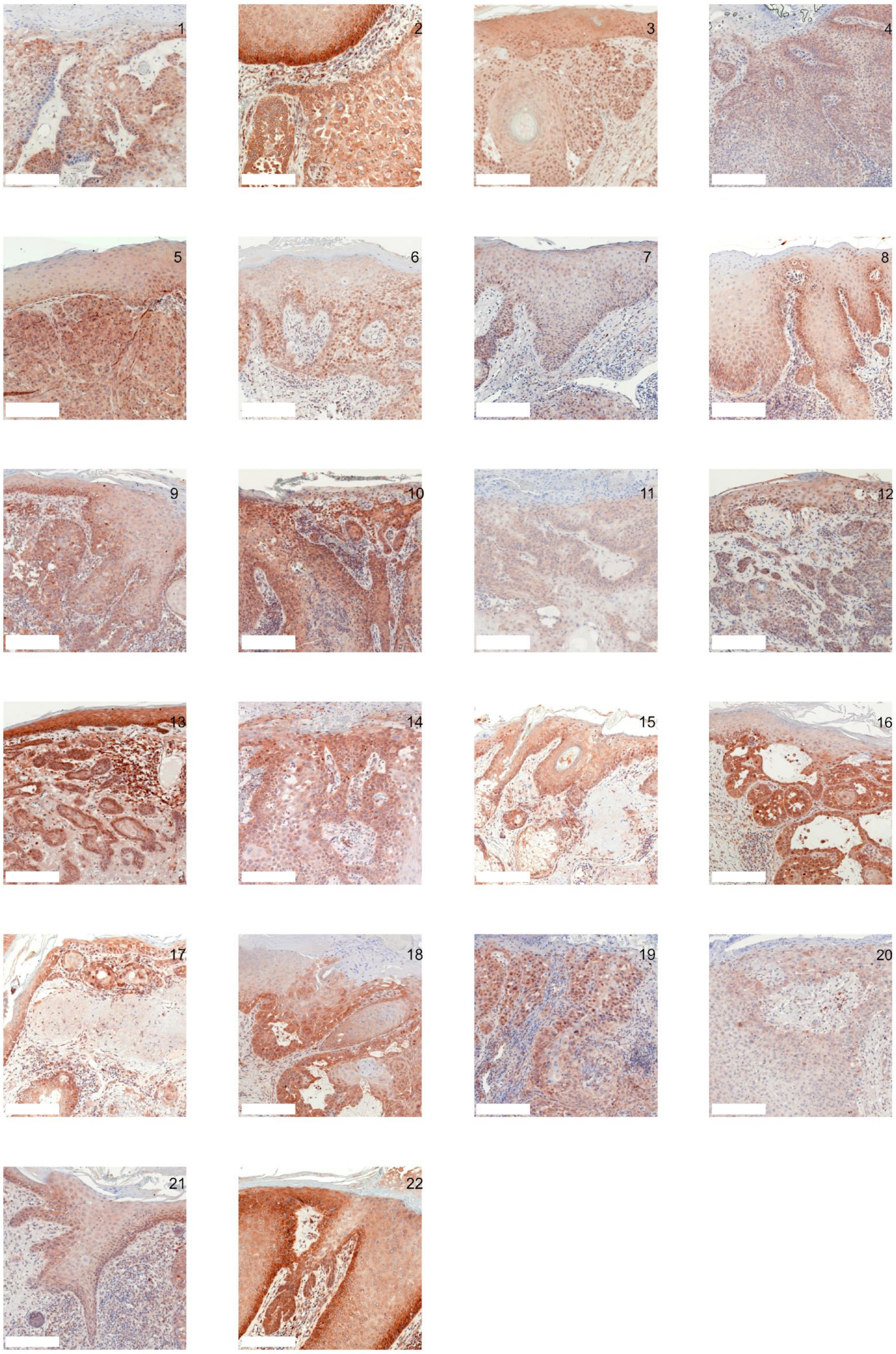

Supplementary Figure S13: Immunohistochemistry for TASK3 of SCC. Scale bars represent 200 µm. SCC showed strong expression in 6/17 and weak expression in 10/17 of tissue samples. Only 1/17 was negative for TASK3. 1 = 1271, 2 = 10173, 3 = 11044, 4 = 1013, 5 = 11190, 6 = 1389, 7 = 2346, 8 = 6837, 9 = 7996, 10 = 8931, 11 = 9314, 12 = 9880, 13 = 9885, 14 = 12597, 15 = 7346, 16 = 2433, [3)]17 = 1279, 18 = 95

[3)] not suitable for evaluation

Supplementary Figure S14. Immunohistochemistry for TASK3 of BCC.

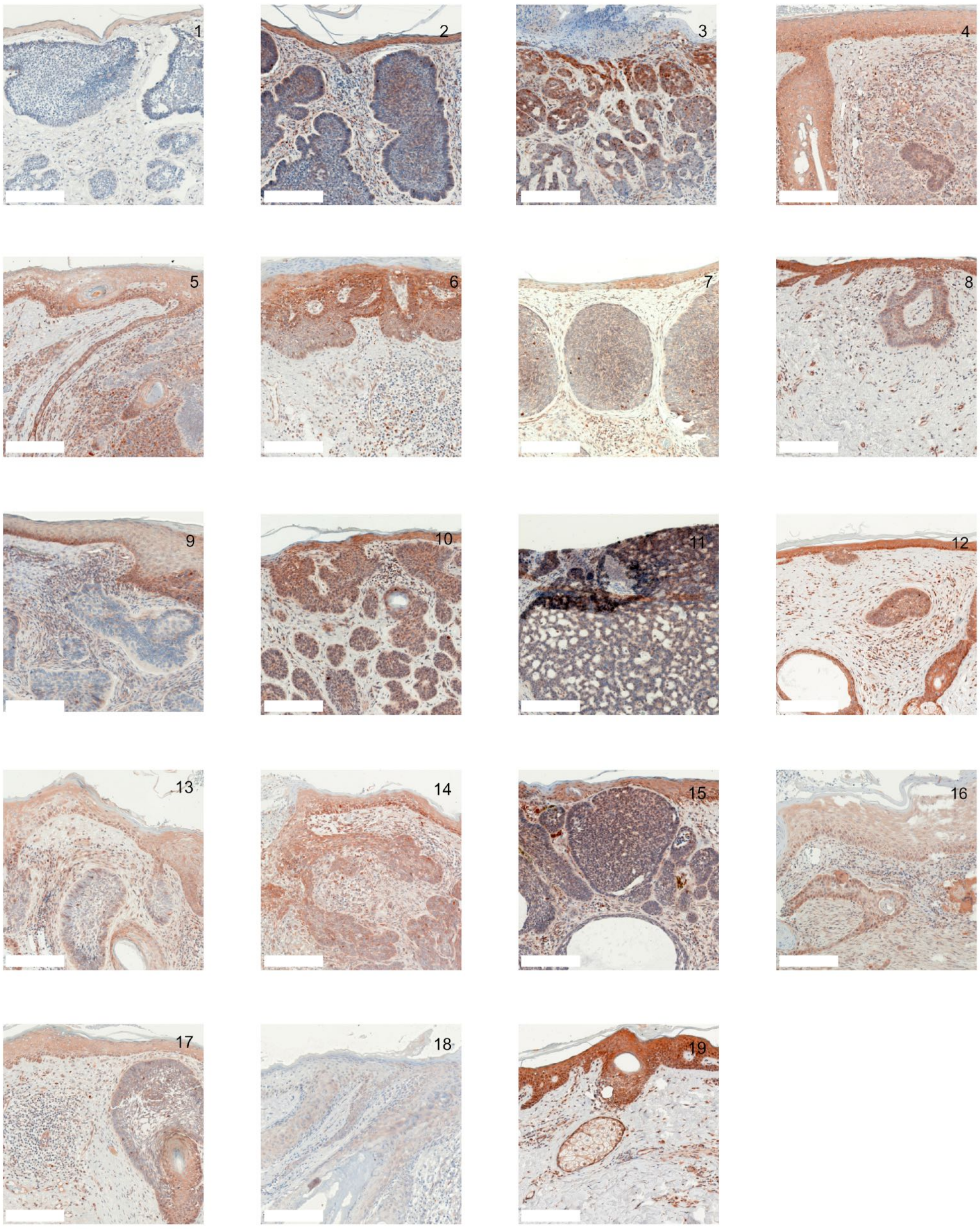

Supplementary Figure S14: Immunohistochemistry for TASK3 of BCC. Scale bars represent 200 µm. 10/18 of BCC showed weak expression of TASK3, whereas 7/18 showed no expression and the remaining 1/18 showed strong expression.

1 = 26, 2 = 27, 3 = 270, 4 = 796, 5 = 816, 6 = 845, 7 = 899, 8 = 911, 9 = 925, 10 = 1000, 11 = 1404, 12 = 1425, 13 = 1904, 14 = 2131, 15 = 999, [3)]16 = 2752, [3)]17 = 1438, 18 = 410, [3)]19 = 1440, [3)]20 = 1425, 21 = 1904

[3)] not suitable for evaluation

Supplementary Figure S15. Immunohistochemistry for TASK3 of NCN.

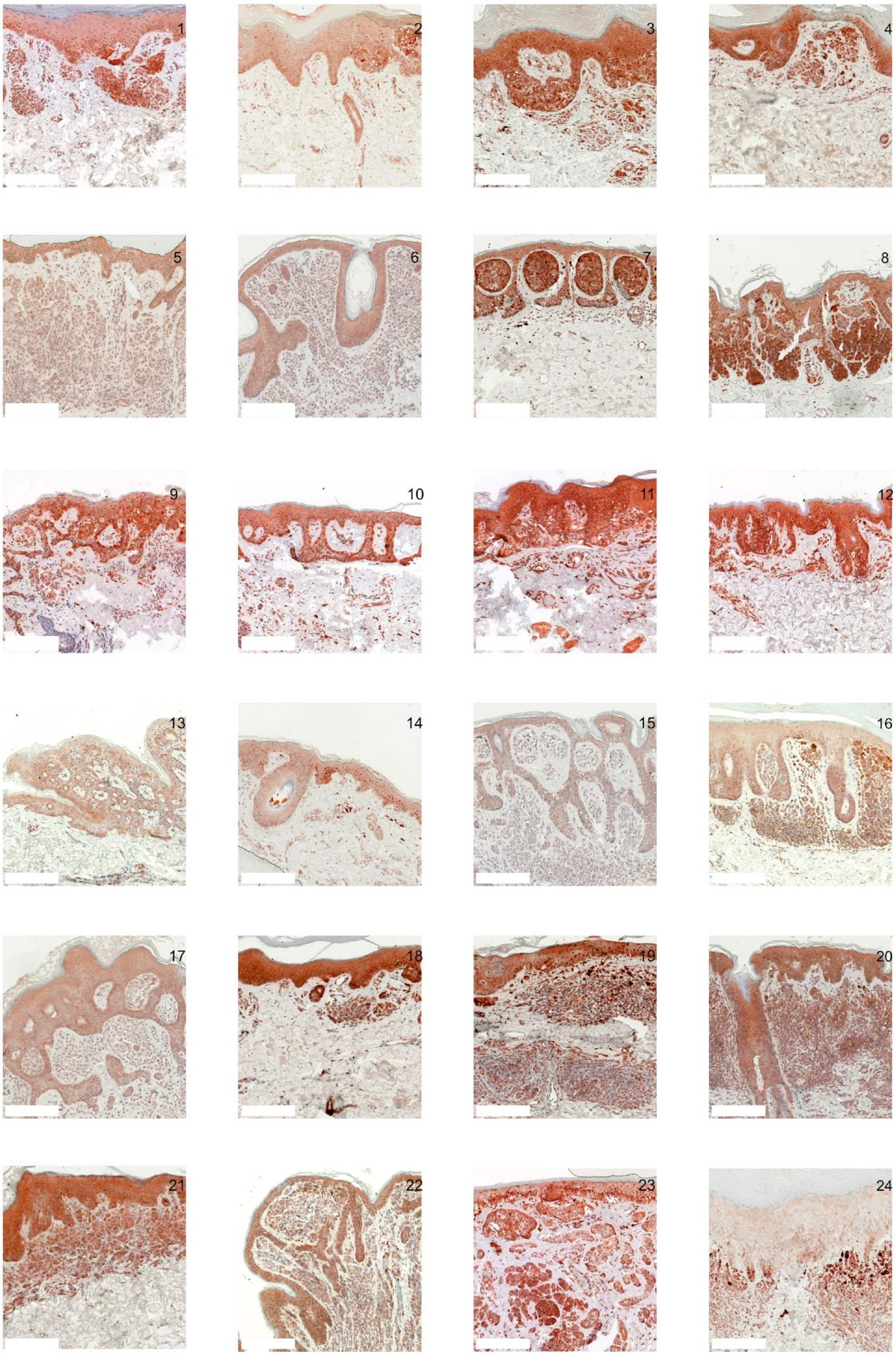

Supplementary Figure S15: Immunohistochemistry for TASK3 of NCN. Scale bars represent 200 µm. In epidermal parts, 6/17 of NCN samples were strong positive, 7/17 weak positive and 4/17 negative for TASK3. Furthermore, dermal sections appeared weak positive in 9/17 NCN. The remaining 8/17 were evenly distributed in negative, respectively strong positive staining results.

1 = 986, 2 = 1054, 3 = 1256, 4 = 1580, 5 = 3795, [1)]6 = 26170, 7 = 26480, [1)]8 = 28814, 9 = 1496, 10 = 2369, 11 = 10092, 12 = 29215, 13 = 5950, 14 = 9285, 15 = 17735, 16 = 19116, 17 = 4251, [1)]18 = 16427, [1)]19 = 1060, [1)]20 = 31892, 21 = 10076, [1)]22 = 18172, 23 = 9625, [1)]24 = 25165

[1)] epidermal part is not expressed or not evaluable

Supplementary Figure S16. Immunohistochemistry for TASK3 of MM.

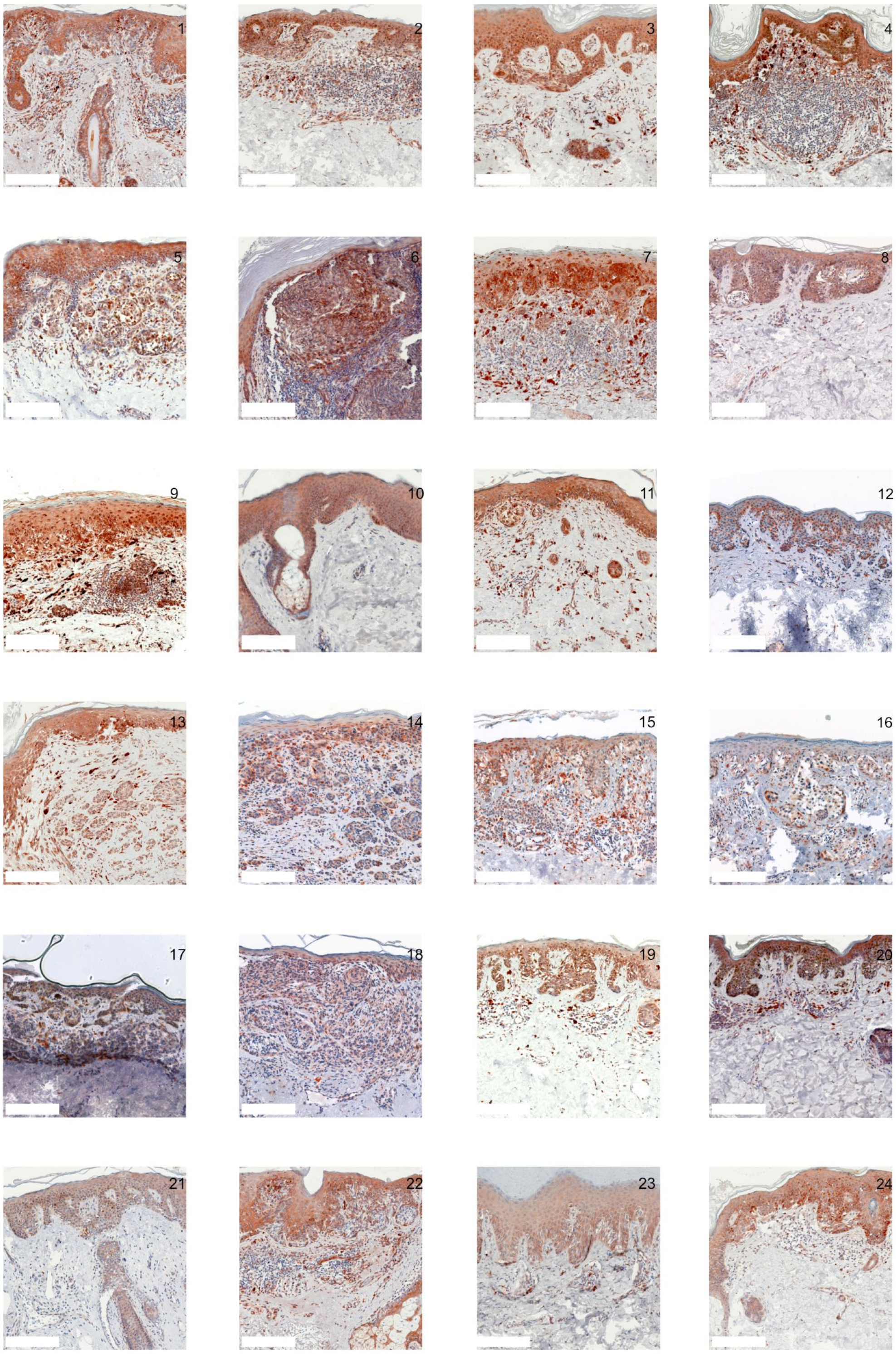

Supplementary Figure S16: Immunohistochemistry for TASK3 of MM. Scale bars represent 200 µm. MM showed strong positive staining in 4/21 epidermal and 5/21 dermal portions. Weak positive staining was seen in 11/21 epidermal and 8/21 dermal portions. Negative results were observed in 6/21 epidermal and 8/21 dermal MM portions.

1 = 730, 2 = 881, [1)]3 = 1316, 4 = 2145, 5 = 5847, 6 = 7781, 7 = 9199, 8 = 9240, 9 = 9255, 10 = 9346, 11 = 9525, [3)]12 = 10891, 13 = 10968, 14 = 7677, 15 = 7802, 16 = 8190, 17 = 9375, 18 = 7060, 19 = 9256, 20 = 2239, [1)]21 = 1109, 22 = 730, 23 = 8740, 24 = 1447

[1)] epidermal part is not expressed or not evaluable

[3)] not suitable for evaluation

Supplementary Figure S17. Tissue Control of Immunohistochemical staining for GPR31.

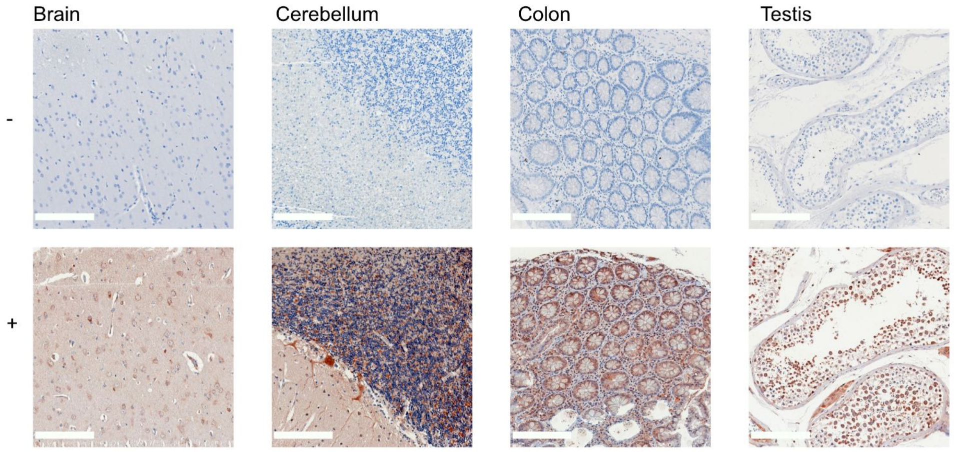

Supplementary Figure S18. Tissue Control of Immunohistochemical staining for GPR151.

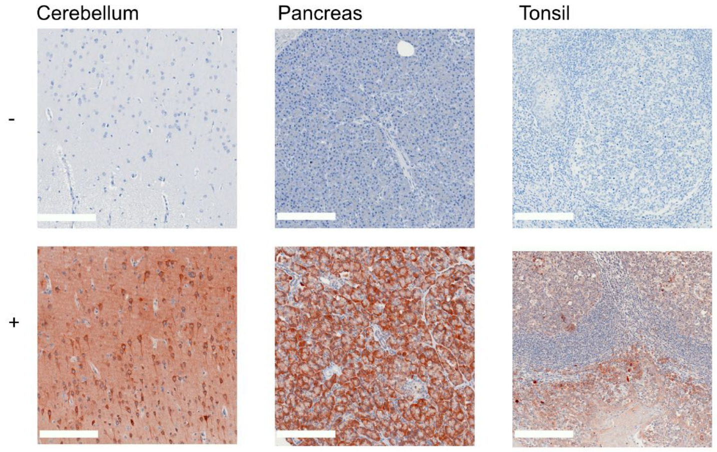

Supplementary Figure S19. Tissue Control of Immunohistochemical staining for TASK1.

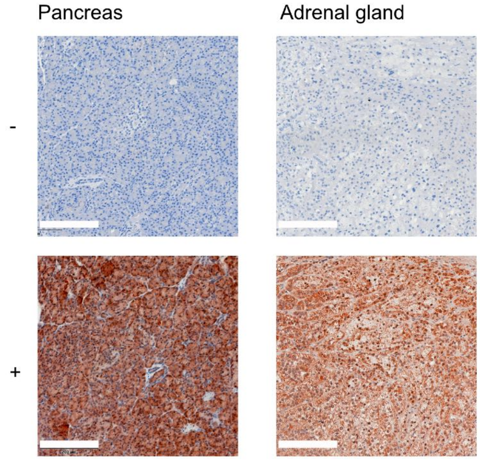

Supplementary Figure S20. Tissue Control of Immunohistochemical staining for TASK3.

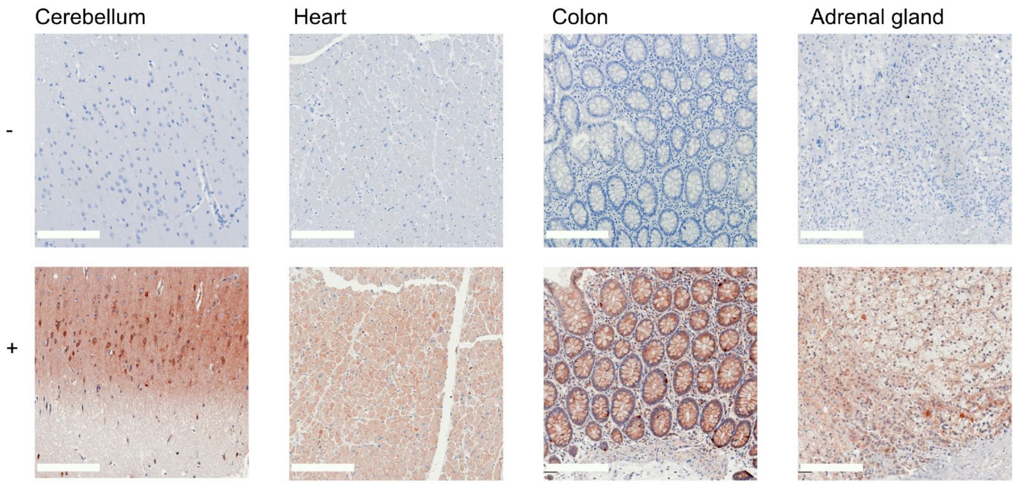

Supplementary Figure S21. Mutation frequencies of GPR31/GPR151/TASK1/TASK3 in non-melanoma skin cancer

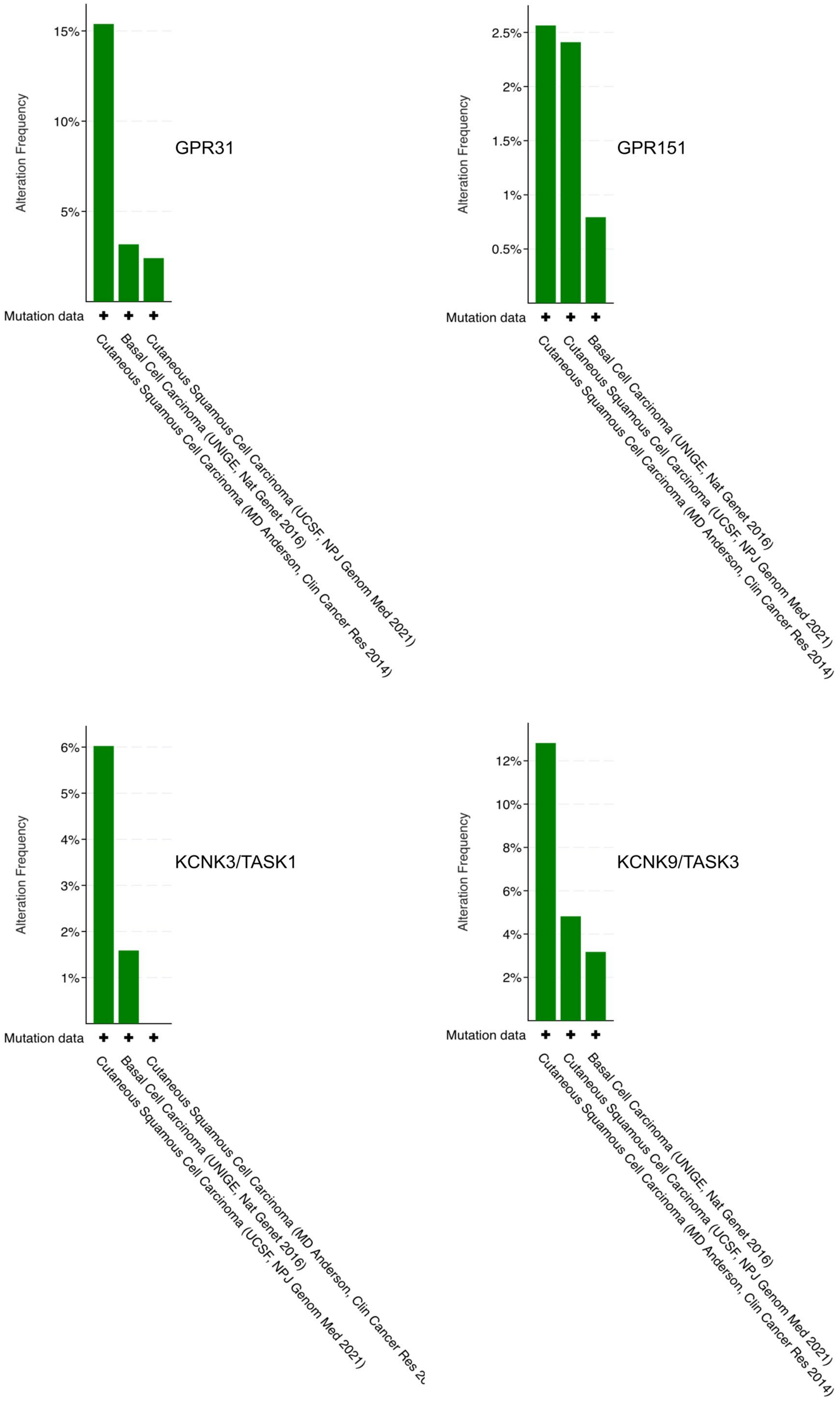

Supplementary Figure S22: Mutation frequencies of GPR31/GPR151/TASK1/TASK3 in MM

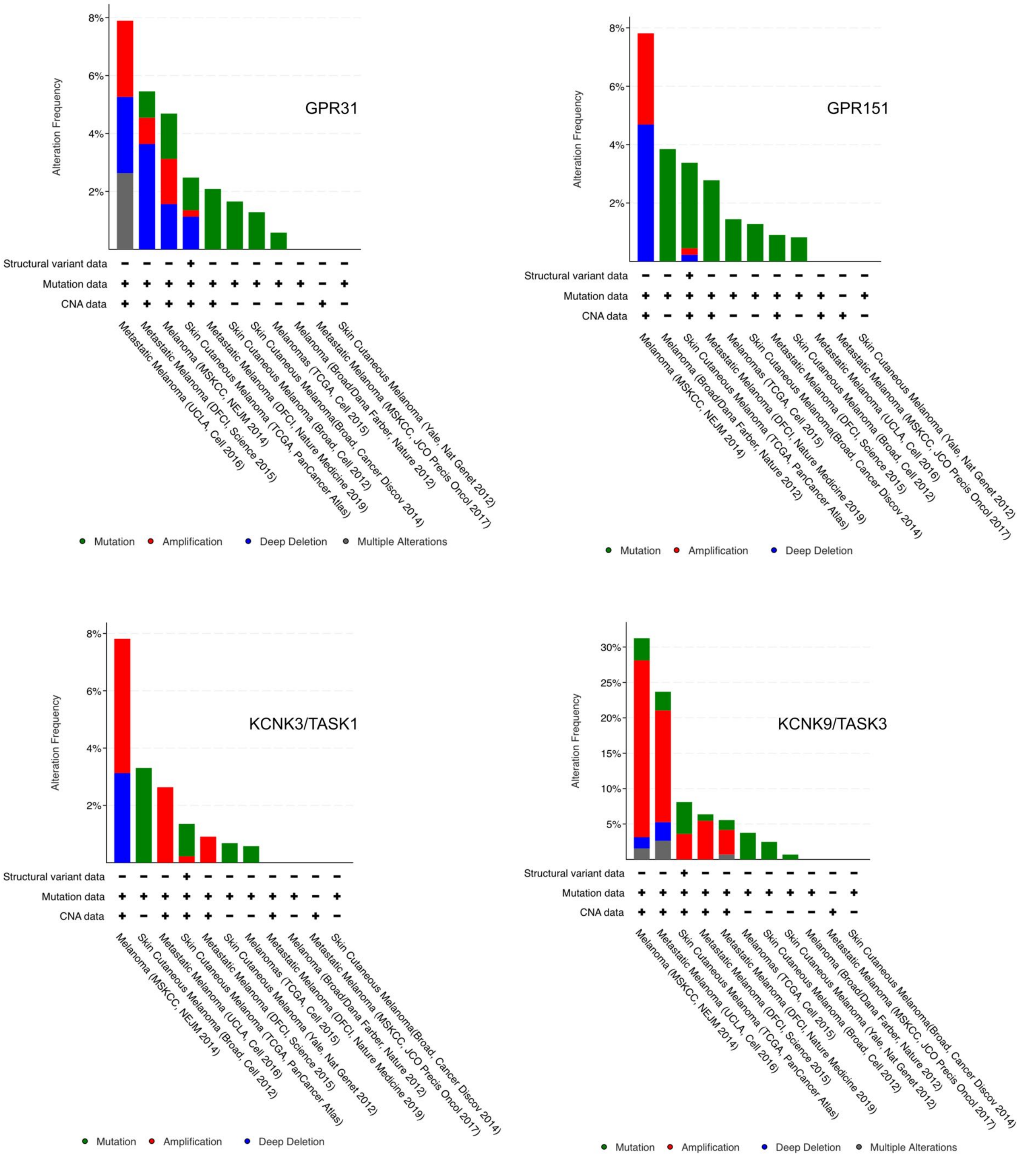

Scoring Tables:

The epidermis was used as a reference structure to determine scoring.

++ for strong positive/positive reaction with >80% of cells positive and/or staining intensity is high

+ for 20–80% of cells with a weak positive/partial positive reaction

- for <20% of cells with weak staining (=negative reaction).

* expression level decreases with increasing tissue depth

** expression level increases with increasing tissue depth

X: not suitable for evaluation

: no tissue available

**Supplementary Table S1** , Scoring for GPR31				
Number	Histo-Number	Sex	Age	Scoring
SCC				
1	9314	m	102	+
2	12791	m	74	+
3	9884	m	74	+
4	11160	m	77	+
5	11049	m	84	+
6	16190	f	45	+
7	9885	m	99	+
8	10979	f	97	+
9	7578	f	84	+
10	8931	f	89	+
11	8196	f	81	+
12	2795	f	87	+
13	9576	m	95	+
14	7973	m	82	+
15	8031	m	101	+
BCC				
1	410	m	98	+
2	27	m	83	-
3	409	m	98	+
4	1589	f	80	+
5	1677	f	81	+
6	1393	m	101	+
7	1587	m	67	+
8	1473	m	91	-
9	837	m	97	-
10	1571	m	88	+
11	1904	f	61	+
12	2131	m	80	+
13	1710	m	95	+
14	1750	m	81	-
15	1393	m	101	-
16	816	f	82	-
17	1048	m	37	-

18	1518	f	37	-	
19	1732	m	83	-	
20	1714	m	79	-	
21	1858	f	77	+	
22	732	m	77	-	
NCN				epidermal	dermal
1	254	m	47	X	+
2	9285-09	m	51	+	-
3	1256	m	28	+	+
4	8134-09	m	58	+	+
5	265	m	73	+	+
6	30	m	72	+	+
7	32	m	41	+	++
8	34	f	45	+	+
9	41	m	54	x	+
10	61	m	75	x	+
11	75	f	52	x	+
12	101	f	59	+	+
13	105	m	33	x	+
14	492	m	53	+	+
15	114	f	71	+	+
16	116	m	28	x	
17	117	m	30	x	
18	121	m	26	x	+
19	122	m	26	+	+
20	496	f	44	x	+
21	154	f	21	x	+
22	156	f	30	+	+
23	223	f	42	+	+
24	244	f	52	+	+
MM				epidermal	dermal
1	6926	m	37	+	+
2	7439	f	52	+	+
3	11094	f	79	+	+
4	9207	f	96	+	+
5	9346	m	68	+	+
6	1345	f	49	+	+
7	881	f	51	+	++
8	9199	f	36	+	+
9	8190	m	68	+	+
10	8718	f	95	+	++
11	7104	m	54	+	+
12	4476	m	82	+	+
13	10968	f	94	+	+
14	11264	f	41	+	++
15	11317	f	57	+	+
16	3341	f	60	+	++
17	7802	m	54	+	++
18	11275	m	84	+	+
19	3679	m	88	++	++
20	7066	m	48	+	+

21	812-11	f	55	+	+
22	757-11	f	69	+	+
23	1817-11	m	66	+	+
24	1903-11	f	54	+	+
25	2017-11	m	56	+	+
26	2125-11	m	79	+	+
27	2668-11	f	43	+	-
28	3065-11	m	91	-	++

**Supplementary Table S2** , Scoring for GPR151					
Number	Histo-Number	Sex	Age	Scoring	
SCC					
1	1271	m	64	+	
2	10173	m	97	+	
3	11044	m	84	+	
4	1013	m	97	+	
5	11190	m	72	+	
6	1389	f	82	++	
7	2346	m	88	-	
8	6837	m	79	+	
9	7996	f	95	++	
10	8931	f	89	++	
11	9314	m	102	++	
12	9880	m	92	++	
13	9885	m	99	+	
14	12597	m	95	+	
15	7346	f	95	++	
16	2433	m	86	+	
17	1279	m	71	x	
18	9576	m	95	+	
BCC					
1	26	m	83	-	
2	27	m	83	-	
3	270	f	72	+	
4	796	f	71	+	
5	816	f	82	-	
6	845	m	97	-	
7	899	m	92	-	
8	911	f	56	-	
9	925	m	94	-	
10	1000	m	97	+	
11	1404	f	72	-	
12	1422	m	80	+	
13	1904	f	61	+	
14	2131	m	80	++	
15	999	m	97	+	
16	2752	m	91	++	
17	722	m	80	+	
18	410	m	98	x	
19	877	m	94	-	
20	1440	f	89	x	
21	265	f	82	+	
NCN				epidermal	dermal
1	986	m	48	++	+ (partially ++)
2	1054	f	63	+	+
3	1256	m	28	+	+
4	1580	m	41	+	+
5	3795	m	26.	+	-

6	26170	f	54	x	+
7	26480	f	57	x	+
8	28814	f	58	+	+
9	1496	f	66	+	+
10	2369	m	80	+	+
11	10092	f	57	+	+
12	29215	f	34	+	+
13	5950	m	53	-	-
14	9285	m	51	-	-
15	17735	m	33	-	-
16	19116	f	37	++	+ *
17	4251	f	69	+	-
18	1060	m	50	x	-
19	27639	m	77	+	+
20	27629	f	53	+	+
21	18172	m	49	-	- (partially +)
MM				epidermal	dermal
1	730	m	77	+	-
2	881	f	51	+	+
3	1316	f	37	-	-
4	2145	m	84	-	- (partially +)
5	5847	f	46	-	-
6	7781	f	80	+	+
7	9199	f	36	+	+
8	9240	m	78	+ (partially -)	+ (partially)-
9	9255	m	94	+	+
10	9346	m	68	+	+ (partially ++)
11	9525	f	30	-	-
12	10891	m	91	+	+
13	10968	f	94	+	+
14	7677	m	88	+	-
15	7802	m	54	+	-
16	8190	m	68	+	+
17	9375	m	64	-	-
18	7060	f	81	+	+
19	9256	m	94	x	x
20	9207	f	96	++	-
21	2239	m	70	+	+
22	11094	f	79	+ ***	+
23	1447	f	57	-	+
24	16846	f	48	+	+

**Supplementary Table S3 ,** Scoring for TASK1					
Number	Histo-Number	Sex	Age		
SCC					
1	1271	m	64	+	
2	10173	m	97	+ (partially ++)	
3	11044	m	84	+ (partially ++)	
4	1013	m	97	+	
5	11190	m	72	+ (partially ++)	
6	1389	f	82	++	
7	2346	m	88	+	
8	6837	m	79	+	
9	7996	f	95	+	
10	8931	f	89	++	
11	9314	m	102	+	
12	9880	m	92	+	
13	9885	m	99	-	
14	12597	m	95	+	
15	7346	f	95	x	
16	2433	m	86	+	
17	1279	m	71	x	
18	9576	m	95	+	
BCC					
1	26	m	83	-	
2	27	m	83	-	
3	270	f	72	+	
4	796	f	71	+	
5	816	f	82	-	
6	845	m	97	+ *	
7	899	m	92	- (partially +)	
8	911	f	56	+	
9	925	m	94	+	
10	1000	m	97	-	
11	1404	f	72	-	
12	1422	m	80	+	
13	1904	f	61	+	
14	2131	m	80	+	
15	999	m	97	+ *	
16	2752	m	91	x	
17	1440	f	89	+	
18	410	m	98	-	
19	1438	f	85	- (partially +)	
20	985	m	80	+	
NCN				Epidermal	dermal
1	986	m	48	+	+
2	1054	f	63	+	+

3	1256	m	28	+	++
4	1580	m	41	+	+
5	3795	m	26	-	-
6	26170	f	54	x	+
7	26480	f	57	+	+
8	28814	f	58	+	+
9	1496	f	66	+	+ *
10	2369	m	80	+	+
11	10092	f	57	+	+
12	29215	f	34	+	+
13	5950	m	53	-	-
14	9285	m	51	+	-
15	17735	m	33	-	+
16	19116	f	37	+ partially ++	+ *
17	4251	f	69	-	-
18	31892	m.	7	x	++ *
19	18172	m.	43	x	+
20	22545	f	80	+	x
21	10076	f	46	-	+
22	16427	f	53	+	+
23	9625	f	33	+	+
24	1060	m	50	x	+
MM				Epidermal	dermal
1	730	m	77	-	-
2	881	f	51	-	+
3	1316	f	37	+	+
4	2145	m	84	-	- (partially +)
5	5847	f	46	-	+
6	7781	f	80	+	+
7	9199	f	36	+	-
8	9240	m	78	-	-
9	9255	m	94	+	+
10	9346	m	68	-	+
11	9525	f	30	-	+***
12	10891	m	91	-	+
13	10968	f	94	+	-
14	7677	m	88	++	++
15	7802	m	54	-	- *
16	8190	m	68	+ ***	+ ***
17	9375	m	64	+	+
18	7060	f	81	+	+
19	9256	m	94	++	-
20	9089	f	66	+	x
21	2293	m	70	x	+
22	9207	f	96	x	
23	1089	m	91	x	
24	11094	f	78	-	x

**Supplementary Table S4 ,** Scoring for TASK3					
Number	Histo-Number	Sex	Age	Scoring	
SCC					
1	1271	m	64	+	
2	10173	m	97	+	
3	11044	m	84	+	
4	1013	m	97	+	
5	11190	m	72	+	
6	1389	f	82	++	
7	2346	m	88	-	
8	6837	m	79	+	
9	7996	f	95	++	
10	8931	f	89	++	
11	9314	m	102	++	
12	9880	m	92	++	
13	9885	m	99	+	
14	12597	m	95	+	
15	7346	f	95	++	
16	2433	m	86	+	
17	1279	m	71	x	
18	9576	m	95	+	
BCC					
1	26	m	83	-	
2	27	m	83	-	
3	270	f	72	++ *	
4	796	f	71	- (partially +)	
5	816	f	82	-	
6	845	m	97	+ *	
7	899	m	92	+	
8	911	f	56	-	
9	925	m	94	-	
10	1000	m	97	+	
11	1404	f	72	+ (partially -)	
12	1425	m	80	+	
13	1904	f	61	-	
14	2131	m	80	+	
15	999	m	97	+	
16	2752	m	91	x	
17	1438	f	85	x	
18	410	m	98	+	
19	1440	f	89	x	
20	1425	m	80		
21	1904	f	82	+	
NCN				Epidermal	dermal
1	986	m	48	++	+
2	1054	f	63	+	+
3	1256	m	28	+	++
4	1580	m	41	+	+
5	3795	m	26	+	-

6	26170	f	54	x	+
7	26480	f	57	++	++
8	28814	f	58	x	++
9	1496	f	66	+	+
10	2369	m	80	+	+
11	10092	f	57	+	+
12	29215	f	34	++	+
13	5950	m	53	-	-
14	9285	m	51	++	+
15	17735	m	33	-	-
16	19116	f	37	++	++
17	4251	f	69	-	-
18	16427	f	53	x	+
19	1060	m	50	x	+ *
20	31892	m	7	x	+
21	10076	f	46	-	++
22	18172	m	43	X	+
23	9625	f	33	++	+ **
24	25165	m.	69	x	++*
MM				Epidermal	dermal
1	730	m	77	x	+
2	881	f	51	++	+ **
3	1316	f	37	x	++*
4	2145	m	84	+	- (partially +)
5	5847	f	46	-	+ ** (partially ++)
6	7781	f	80	+	+ (partially ++)
7	9199	f	36	++	++
8	9240	m	78	-	-
9	9255	m	94	++	++
10	9346	m	68	-	++
11	9525	f	30	+	++
12	10891	m	91	x	x
13	10968	f	94	-	- (partially ++)
14	7677	m	88	+	+*
15	7802	m	54	-	-
16	8190	m	68	+	++ *
17	9375	m	64	+	+
18	7060	f	81	++	-
19	9256	m	94	+	+
20	2239	m	70	+	-
21	11094	f	79	x	+
22	730	m	77	-	-

23	8740	m	56	+	-
24	1447	f	57	+***	+

Table S5: Comprehensive data on scoring results for SCC:

Number	GPR31	GPR151	TASK1	TASK3
1271	x	+	+	+
10173	x	+	+ (partially ++)	+
11044	x	+	+ (partially ++)	+
1013	x	+	+	+
11190	x	+	+ (partially ++)	+
1389	x	++	++	++
2346	x	-	+	-
6837	x	+	+	+
7996	x	++	+	++
8931	+	++	++	++
9314	+	++	+	++
9880	x	++	+	++
9885	+	+	-	+
12597	x	+	+	+
7346	x	++	x	++
2433	x	+	+	+
1279	x	x	x	x
9576	+*	+	+	+
12791	+			
9884	+			
11160	+			
11049	+			
16190	+			
10979	+			
7578	+			
8196	+			
2795	+			
9576	+			
7973	+			
8031	+			

Table S6: Comprehensive data on scoring results for BCC:

Number	GPR31	GPR151	TASK1	TASK3
26		-	-	-
27	-	-	-	-
270		+	+	++ *
796		+	+	- (partially +)
816	-	-	-	-
845		-	+ *	+ *
899		-	- (partially +)	+
911		-	+	-
925		-	+	-
1000		+	-	+
1404		-	-	+ (partially -)
1422		+	+	+
1904	+	+	+	-
2131	+	++	+	+
999		+	+ *	+
2752		++	x	x
1438		x	- (partially +)	x
410	+	x	-	+
1440		x	+	x
1425		x	x	x
1904		x	x	+
985		x	+	x
722		+		
877		-		
265		+		
409	+			
1589	+			
1677	+			
1393	+			
1587	+			
1473	-			
837	-			
1571	+			
1710	+			
1750	-			
1393	-			
1048	-			
1518	-			
1732	-			
1714	-			
1858	+			
732	-			

Table S7: Comprehensive data on scoring results for NCN

Number	GPR31		GPR151		TASK1		TASK3	
	Epidermal	Dermal	Epidermal	Dermal	Epidermal	Dermal	Epidermal	Dermal
986			++	+ (partially ++)	+	+	++	+
1054			+	+	+	+	+	+
1256	+	+	+	+	+	++	+	++
1580			+	+	+	+	+	+
3795			+	-	-	-	+	-
26170			x	+	x	+	x	+
26480			x	+	+	+	++	++
28814			+	+	+	+	x	++
1496			+	+	+	+ *	+	+
2369			+	+	+	+	+	+
10092			+	+	+	+	+	+
29215			+	+	+	+	++	+
5950			-	-	-	-	-	-
9285	+	-	-	-	+	-	++	+
17735			-	-	-	+	-	-
19116			++	+ *	+ partially ++	+ *	++	++
4251			+	-	-	-	-	-
16427			x	x	+	+	x	+
1060			X	-	x	+	x	+ *
31892			x	x	X	++*	x	+
10076			x	x	-	+	-	++
18172			-	-	x	+	X	+
9625			x	x	+	+	++	+ **
25165			x	x	x	x	x	++*
22545			x	x	+	x		
27639			+	+	+	+		
27629			+	+	+	+		
254	X	+						
8134-09	+	+						
265	+	+						
30	+	+						
32	+	++						
34	+	+						
41	x	+						
61	x	+						
75	x	+						
101	+	+						
105	x	+						
492	+	+						
114	+	+						
116	x	x						
117	x	x						
121	x	+						
122	+	+						
496	x	+						
154	x	+						
156	+	+						
223	+	+						
244	+	+						

Table S8: Comprehensive data on scoring results for MM:

Number	GPR31		GPR151		TASK1		TASK3	
	Epidermal	Dermal	Epidermal	Dermal	Epidermal	Dermal	Epidermal	Dermal
6926	+	+						
7439	+	+						
11094	+	+						
9207	+	+						
9346	+	+						
1345	+	+						
881	+	++						
9199	+	+						
8190	+	+						
8718	+	++						
7104	+	+						
4476	+	+						
11264	+	++						
11317	+	+						
3341	+	++						
7802	+	++						
11275	+	+						
3679	++	++						
7066	+	+						
812-11	+	+						
757-11	+	+						
1817-11	+	+						
1903-11	+	+						
2017-11	+	+						
2125-11	+	+						
2668-11	+	-						
3065-11	-	++						
730			+	-	-	-	+	+
881			+	+	-	+	++	+ **
1316			-	-	+	+	x	++*
2145			-	- (partially +)	-	- (partially +)	+	- (partially +)
5847			-	-	-	+	-	+ ** (partially ++)
7781			+	+	+	+	+	+ (partially ++)
9199			+	+	+	-	++	++
9240			+ (partially -)	+ (partially -)	-	-	-	-
9255			+	+	+	+	++	++
9346			+	+ (partially ++)	-	+	-	++
9525			-	-	-	+***	+	++
10891			+	+	-	+	x	x
10968	+	+	+	+	+	-	-	- partially ++
7677			+	-	++	++	+	+*

7802			+	-	-	- *	-	-
8190			+	+	+ ***	+ ***	+	++ *
9375			-	-	+	+	+	+
7060			+	+	+	+	++	-
9256					++	-	+	+
9207			++	-	x	x	X	x
2239			+	+	X	+	+	-
11094			+ ***	+	-	x	X	+
1447			-	+	x	x	+***	+
16846			+	+	x	x	x	x
9089					+	x		
1089					x	x		
8740							+	-

Table S9: Statistical analysis – comparison of all entities

Pairwise Comparisons of all entities					
NCN and MM epidermal portions			NCN and MM dermal portions		
type	Pairs	p-value	type	Pairs	p-value
GPR31	BCC-MM	0.000**	GPR31	BCC-MM	0.001**
	BCC-SCC	0.000**		BCC-SCC	0.002*
	BCC-NCN	0.000**		BCC-NCN	0.000**
	MM-SCC	0.966		MM-SCC	0.986
	MM-NCN	0.966		MM-NCN	0.131
	SCC-NCN	1.000		SCC-NCN	0.184
GPR151	BCC-MM	0.386	GPR151	BCC-MM	0.733
	BCC-SCC	0.002**		BCC-SCC	0.002**
	BCC-NCN	0.189		BCC-NCN	0.984
	MM-SCC	0.013*		MM-SCC	0.004**
	MM-NCN	0.603		MM-NCN	0.735
	NCN-SCC	0.064		NCN-SCC	0.001**
TASK1	BCC-MM	0.934	TASK1	BCC-MM	0.045*
	BCC-SCC	0.011*		BCC-SCC	0.008**
	BCC-NCN	0.304		BCC-NCN	0.496
	MM-SCC	0.012*		MM-SCC	0.392
	MM-NCN	0.332		MM-NCN	0.186
	NCN-SCC	0.111		NCN-SCC	0.042*
TASK3	BCC-MM	0.279	TASK3	BCC-MM	-
	BCC-SCC	0.008**		BCC-SCC	-
	BCC-NCN	0.053		BCC-NCN	-
	MM-SCC	0.090		MM-SCC	-
	MM-NCN	0.353		MM-NCN	-
	NCN-SCC	0.463		NCN-SCC	-

Results of testing with Kruskal-Wallis test and post-hoc Bonferroni comparison.

*p<0.05 = significant, **p<0.01 = highly significant. Marked in red.

- means not applicable as values were exactly the same between NCN and MM for TASK3

Table S10: Statistical analysis – separate comparison: non-melanoma skin cancer and melanocytic tumors

BCC vs. SCC	
GPR31	0.009**
GPR151	0.009**
TASK1	0.029*
TASK3	0.011*

Values for Mann-Whitney U test results for exact significance shown as [2*(1-tailed Significance), not corrected for ties]. *p<0.05 = significant, **p<0.01 = highly significant.

MM vs. NCN		
	Epidermal	Dermal
GPR31	1.000	0.071
GPR151	0.563	0.693
TASK1	0.328	0.193
TASK3	0.424	-

Values for Mann-Whitney U test results. Based on normal approximation (dermal, asymptotic significance (2-tailed) or exact significance [epidermal, 2*(1-tailed Significance), not corrected for ties]. *p<0.05 = significant, **p<0.01 = highly significant.

Table S11: NCBI Geo Gene Expression Profile Analysis

BCC vs. SCC: Study GSE53462

ID	adj.P.Val	P.Value	t	B	logFC	Gene.symbol	Gene.title
**ILMN_1679962**	0.712877	3.69e-01	0.9171575	-5.78096	0.04335712	GPR31	G protein-coupled receptor 31
**ILMN_1811039**	0.513024	1.67e-01	1.4325979	-5.21309	0.08212953	GPR151	G protein-coupled receptor 151
**ILMN_1768483**	0.748612	4.22e-01	-0.8185928	-5.86382	-0.06563691	KCNK3	potassium two pore domain channel subfamily K member 3
**ILMN_1664919**	0.533884	1.82e-01	1.3789673	-5.28207	0.09579275	KCNK9	potassium two pore domain channel subfamily K member 9

NCN vs. MM: Study GSE4587

ID	adj.P.Val	P.Value	t	B	logFC	Gene.symbol	Gene.title
**208556_at**	0.96986	9.28e-01	0.092896	-6.218245	0.0562666	GPR31	G protein-coupled receptor 31
**228127_at**	0.45822	2.00e-01	1.368699	-5.318113	0.5948482	KCNK3	potassium two pore domain channel subfamily K member 3
**238870_at**	0.46982	2.10e-01	-1.3373853	-5.355781	-1.3248295	KCNK9	potassium two pore domain channel subfamily K member 9

NCN vs. MM: Study GSE12391

ID	adj.P.Val	P.Value	t	B	logFC	Gene.symbol	Gene.title
**43277**	0.346887	9.48e-02	-1.7082155	-4.70419	-0.06863678	GPR31	G protein-coupled receptor 31
**34460**	0.442861	1.59e-01	-1.4326781	-5.0997	-0.06456511	KCNK3	potassium two pore domain channel subfamily K member 3
**39399**	0.068094	2.82e-03	-3.1688744	-1.72063	-0.12247674	KCNK9	potassium two pore domain channel subfamily K member 9

NCN vs. MM: Study GSE46517

ID	adj.P.Val	P.Value	t	B	logFC	Gene.symbol	
**208556_at**	5.63e-01	2.57e-01	1.148443	-5.659543	0.5542371	GPR31	G protein-coupled receptor 31
**205952_at**	3.40e-01	9.95e-02	1.68429	-4.944417	0.3012486	KCNK3	potassium two pore domain channel subfamily K member 3

NCN vs. MM: Study GSE114445

ID	adj.P.Val	P.Value	t	B	logFC	Gene.symbol	Gene.title
**208556_at**	0.79786681	6.08e-01	-0.5208198	-6.14076	-0.03911069	GPR31	G protein-coupled receptor 31
**205952_at**	0.41567365	1.69e-01	1.425812	-5.2973417	0.1712021	KCNK3	potassium two pore domain channel subfamily K member 3
**224072_s_at**	0.83493704	6.68e-01	0.4349084	-6.1818071	0.03846382	KCNK9	potassium two pore domain channel subfamily K member 9

NCN vs. MM: Study GSE183115

ID	adj.P.Val	P.Value	t	B	logFC	Gene.Symbol	
**ADXECRS.36994_at**	891	0.15151987	-1.55	-4.71826	-2.27e-01	GPR31	G protein-coupled receptor 31
**ADXECRS.34899_at**	839	0.06915123	2.04	-4.08388	3.60e-01	GPR151	G protein-coupled receptor 151
**ADXEC.31259.C1_at**	912	0.22257079	-1.30	-5.01148	-1.16e-01	KCNK3	potassium two pore domain channel subfamily K member 3
**ADXECRS.30408_at**	915	0.23582718	1.26	-5.05407	1.23e-01	KCNK9	potassium two pore domain channel subfamily K member 9
